# Melatonin beyond chronobiology: integrating pharmacokinetics, dose-exposure forecasting, and clinical evidence

**DOI:** 10.1007/s11033-026-12765-z

**Published:** 2026-09-22

**Authors:** Sergio Pandolfi, Francesco Maria Paone, Marco Metalla, Charlye Ghezzi, Geir Bjørlund, Salvatore Chirumbolo

**Affiliations:** 1 High Master School of Oxygen-Ozone Therapy, University of Pavia, Pavia, Italy; 2 Department of Paediatrics, University of Tor Vergata, Rome, Italy; 3 Istituto Farmacologico Italiano, Milan, Italy; 4 Council for Nutritional and Environmental Medicine (CONEM), Mo i Rana, Norway; 5 Department of Engineering for Innovation Medicine, University of Verona, Strada Le Grazie 8, Verona, 37134 Italy

**Keywords:** Melatonin, Oral, Intravenous, Pharmacokinetics, Review

## Abstract

Melatonin is established as a regulator of circadian timing and sleep, while a wider range of receptor-dependent and receptor-independent biological effects has generated interest in additional therapeutic applications. This review critically examines whether those proposed effects can be interpreted within an exposure-based pharmacological framework. We integrate recent preclinical and clinical evidence with the pharmacokinetics of oral and intravenous melatonin and with ordinary differential equation (ODE)-based dose-exposure forecasting. Oral melatonin is rapidly absorbed but undergoes extensive first-pass metabolism and shows substantial interindividual variability, whereas intravenous administration produces higher and more predictable systemic concentrations. These pharmacokinetic differences do not imply therapeutic superiority of the intravenous route. Rather, they highlight the need to distinguish established chronobiotic uses from investigational high-exposure pharmacology. Clinical evidence is most coherent for sleep and circadian outcomes; evidence for perioperative, analgesic, cardiovascular, renal, anti-inflammatory, neuroprotective, and other non-circadian applications is more heterogeneous and frequently lacks pharmacokinetic-pharmacodynamic measurements. The modelling presented here is therefore hypothesis-generating and is intended to relate dose to exposure, not to provide validated treatment recommendations. Future studies should prospectively link administered dose, timing, formulation, achieved concentration, target engagement, and clinically meaningful outcomes.

## Key points

Melatonin is best established as a chronobiotic agent; proposed non-circadian effects require indication-specific interpretation rather than extrapolation from biological pleiotropy.

Oral and intravenous melatonin should be viewed as pharmacokinetically distinct routes, not as a therapeutic hierarchy: oral administration has the stronger clinical evidence base, whereas intravenous administration remains investigational and mainly illustrates the consequences of controlled high systemic exposure.

Future studies should link dose, formulation, route, timing, achieved exposure, target engagement, and clinical outcome instead of treating nominal melatonin dose as a transferable measure across indications.

## Introduction

Melatonin is an indoleamine best established for its role in circadian organization and sleep regulation, although experimental and clinical research has extended its pharmacological relevance beyond chronobiology [1–6]. These additional actions involve receptor-dependent signaling and receptor-independent modulation of redox homeostasis, mitochondrial function, inflammatory signaling, and cellular stress responses [2, 4–6]. However, biological pleiotropy does not imply universal therapeutic efficacy; its clinical relevance must be considered in relation to indication, dose, timing, formulation, route, and achieved exposure.

Melatonin is also synthesized outside the pineal gland, including in the retina, gastrointestinal tract, bone marrow, lymphocytes, skin, and reproductive tissues, supporting both systemic and local signaling functions [2, 3, 7, 8]. Clinical investigation has consequently expanded from sleep and circadian disorders to perioperative medicine, critical care, cardiovascular disease, inflammation, and pain [9–14]. Evidence across these domains is heterogeneous, emphasizing the need to distinguish mechanistic plausibility from demonstrated clinical benefit.

A central issue is the marked route-dependent difference in melatonin exposure. Oral administration is clinically established, particularly for sleep and circadian indications, but extensive first-pass metabolism results in low and variable bioavailability. Intravenous administration bypasses first-pass metabolism and produces substantially higher and more predictable systemic exposure; however, this pharmacokinetic advantage does not establish superior clinical efficacy, and IV melatonin remains comparatively underexplored [15, 16]. Oral exposure is further influenced by gastrointestinal absorption, CYP-mediated metabolism, age, smoking, caffeine, oral contraceptives, comorbidity, critical illness, inflammation, and concomitant medications [15–21]. Consequently, nominal dose alone may be an unreliable surrogate for systemic exposure.

Human IV pharmacokinetic studies illustrate this route-dependent contrast. A 10-mg IV dose produces substantially higher peak concentrations than oral administration, followed by rapid elimination, with a volume of distribution of approximately 1.2 L/kg and clearance near 0.022 L/min/kg [15, 22–24]. Similarly, 10- and 100-mg IV doses produce supraphysiological but rapidly declining concentrations [22]. Although these doses were not associated with measurable psychomotor impairment or major acute adverse effects in controlled healthy-volunteer studies [15, 22], such observations cannot be generalized to different patient populations, neurological disorders, repeated administration, or prolonged exposure.

The analgesic literature illustrates the broader difficulty of translating mechanistic effects into clinical efficacy. Experimental studies implicate MT1/MT2 receptors, opioid and GABAergic systems, NMDA signaling, ion channels, redox pathways, and inflammatory mediators [25–29], whereas human findings are inconsistent. Notably, a controlled inflammatory pain study found no significant analgesic, anti-hyperalgesic, or peripheral anti-inflammatory effect following 10 or 100 mg IV melatonin [30]. Thus, mechanistic plausibility and high systemic exposure cannot themselves predict clinical benefit.

Accordingly, the relevant translational question is not whether melatonin influences multiple biological processes, but whether a defined dose, administered by an appropriate route and at an appropriate biological time, achieves sufficient exposure to engage a specified target and improve a clinically meaningful endpoint. Oral and IV administration are therefore considered here as pharmacokinetically distinct exposure conditions rather than as a therapeutic hierarchy.

**Fig. 1:**
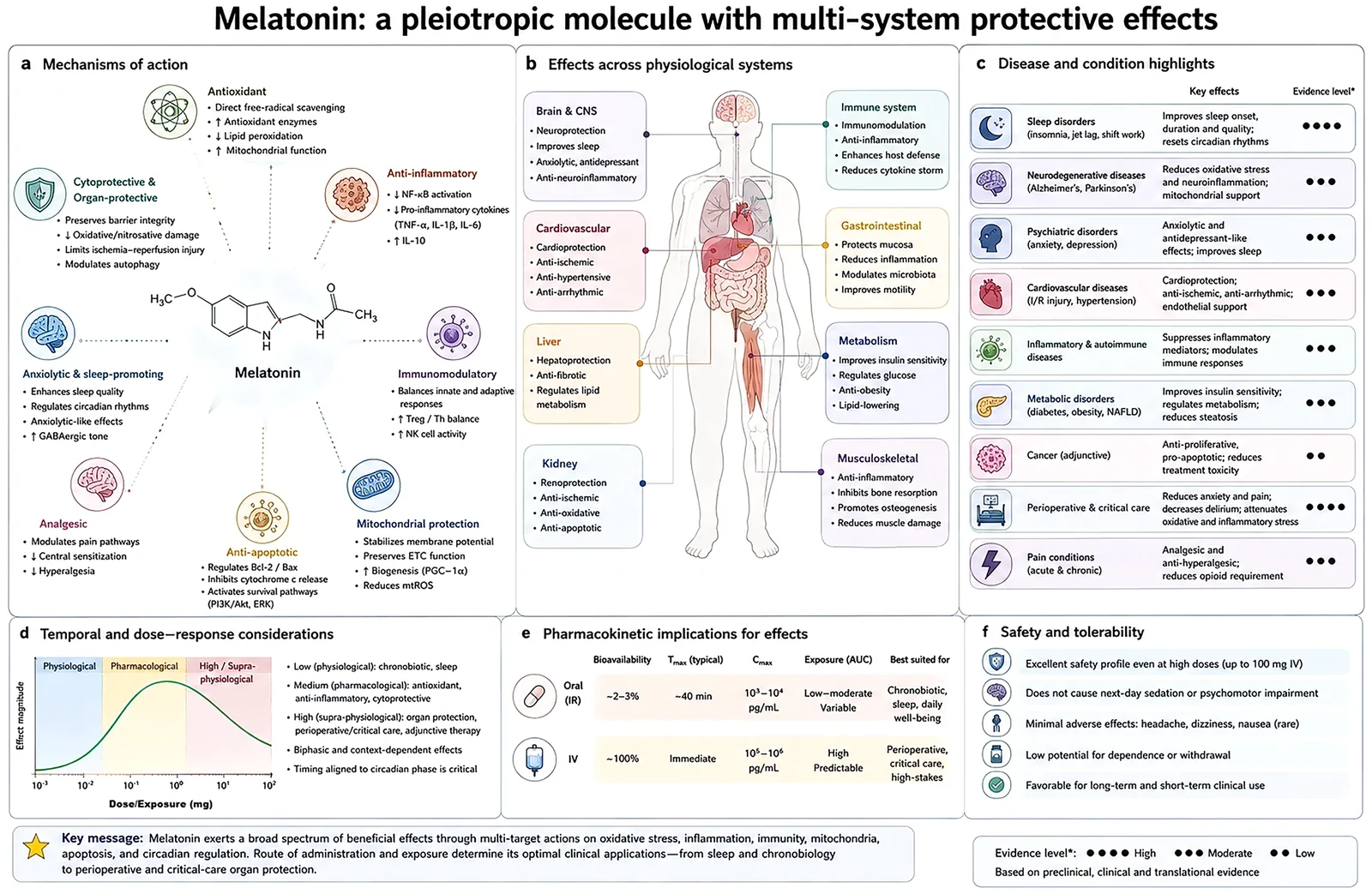
Exposure- and context-dependent interpretation of melatonin biology. The figure summarizes established and proposed actions of melatonin while emphasizing that biological pleiotropy does not imply universal therapeutic benefit. Panel (a) depicts major mechanistic domains, including circadian signaling, redox regulation, mitochondrial function, inflammatory signaling, apoptosis-related pathways, and neuromodulation. Panel (b) indicates organ systems in which these mechanisms have been investigated. Panel (c) distinguishes established chronobiotic applications from emerging or investigational clinical domains. Panel (d) presents a conceptual exposure-response framework in which physiological/chronobiotic effects and higher pharmacological exposures are treated as distinct questions rather than interchangeable dose ranges. Panel (e) compares oral and IV pharmacokinetics without implying therapeutic superiority of either route. Panel (f) emphasizes that tolerability and biological effects are dose-, timing-, population-, and disease-context dependent; a generally favourable safety profile at commonly studied doses should not be generalized to all neurological or high-exposure settings. Figures were prepared using Python (version 3.15; Python Software Foundation), including SciPy and Matplotlib, R (version 3.11.1; R Foundation for Statistical Computing), and Stata version 19 (StataCorp LLC)

This review integrates preclinical mechanisms, human clinical evidence, route-specific pharmacokinetics, and ODE-based dose–exposure forecasting to identify where biological mechanisms, systemic exposure, and clinical findings converge or remain disconnected. Particular emphasis is placed on distinguishing established chronobiotic use from emerging pharmacological hypotheses and on defining exposure–response relationships requiring empirical validation. Figure 1 summarizes this exposure- and context-dependent framework.

### Literature search

A focused PubMed-based literature assessment was used to characterize the breadth of melatonin research and to support the narrative and modelling components of this review. A focused PubMed-based literature assessment was undertaken to support the narrative and exploratory scientometric components of this review. The search term ‘melatonin’ retrieved 37,072 records at the time of assessment. A dataset of 1,076 PMID-indexed records was subsequently used for exploratory scientometric analyses of publication year, article type, journal, authorship, keywords, MeSH terms, and abstract content. Because this was not a systematic review and the dataset was not designed as a probability sample of the complete melatonin literature, the scientometric findings should be interpreted descriptively and should not be assumed to provide exhaustive or statistically representative coverage of the field.

Because the present work is not a formal systematic review, these counts are descriptive and should not be interpreted as evidence of exhaustive capture across all databases.

The 1,076-record PMID dataset was used to examine publication year, article type, journal, authorship, keywords, MeSH terms, and abstract content. These variables support descriptive publication curves, thematic mapping, article-type distributions, and keyword co-occurrence analyses. The scientometric component is intended to contextualize the maturity and thematic breadth of the field rather than to establish therapeutic efficacy.

For temporal growth, the main mathematical model is the logistic ordinary differential equation (ODE):

$$\:\frac{dN}{dt}=rN(1-\frac{N}{K})$$

where N(t) represents the cumulative number of melatonin publications at time t, r is the intrinsic scientific growth rate, and K is the estimated saturation level or carrying capacity of the field. A logistic ODE was explored as one possible phenomenological description of cumulative publication growth. Its parameters should not be interpreted as demonstrating that melatonin research has a true biological or scientific ‘carrying capacity’; rather, the model provides a parsimonious description of a potentially saturating trajectory that can be compared with alternative growth specifications.

The corresponding logistic solution is:

$$\:N\left(t\right)=\frac{K}{1+{e}^{-r(t-{t}_{0})}}$$

where t_0_ is the inflection year, representing the point at which the field reaches its maximum growth velocity. For comparison, an exponential model may also be tested:

$$\:N\left(t\right)={N}_{0}{e}^{rt}$$

but this should be interpreted cautiously, because exponential models assume unlimited growth and may overestimate future publication volume.

The logistic model is

$$\:N\left(t\right)=\frac{K}{1+\mathrm{e}\mathrm{x}\mathrm{p}[-r(t-{t}_{0})}$$.

We fitted both models to the same years and same cumulative publication data, preferably by nonlinear least squares. Then we compared at least:

$$\:\:RMSE=\sqrt[]{\frac{1}{n}}\sum\:_{i=1}^{n}({N}_{i}-{\dot{N}}_{i})$$

and

$$\:AIC=n\:\mathrm{l}\mathrm{n}(RSS/n)+2k$$.

The useful comparison is

$$\:{\varDelta\:AIC}_{c}={AIC}_{c,model}-\mathrm{m}\mathrm{i}\mathrm{n}\left({AIC}_{c}\right)$$.

As a practical interpretation, ΔAIC_c_ <2 means little separation; 4–7 suggests appreciably less support; >10 indicates substantially less support for that model. We did not select logistic simply because its R^2^ is higher, the extra parameter makes that almost inevitable in some datasets.

For thematic evolution, each research topic can be modelled dynamically as:

$$\:\frac{{dT}_{i}}{dt}={a}_{i}{T}_{i}-{b}_{i}{T}_{i}+\sum\:_{j}{c}_{ij}{T}_{j}$$

where T_i_ is the size of topic i, a_i_ is its growth rate, b_i_ is its decline or obsolescence rate, and c_ij_ represents interaction or transition from topic j to topic i. This framework can describe how themes such as sleep medicine, oxidative stress, oncology, inflammation, neurology, reproduction, perioperative care, and critical care expand, contract, or interact over time.

The analysis proceeded in four stages: retrieval of the PMID metadata, generation of descriptive bibliometric indicators, fitting of temporal growth models to annual and cumulative publication counts, and thematic mapping using keywords, MeSH terms, and abstracts.

These bibliometric and ODE-based growth models are exploratory tools for describing the evolution of melatonin research. They should not be interpreted as substitutes for systematic evidence synthesis or as precise forecasts of future publication activity.

Therefore, cumulative annual publication counts were fitted using exponential and logistic growth models over the same observation period. Parameters were estimated by nonlinear least squares. Model fit was evaluated using the residual sum of squares (RSS), root-mean-square error (RMSE), and coefficient of determination (R^2^). Because the candidate models differed in the number of estimated parameters, relative model support was assessed using the small-sample corrected Akaike information criterion (AIC_c_) and ΔAIC_c_. Residuals were additionally inspected for systematic temporal structure. The logistic model was interpreted as a phenomenological description of potentially saturating publication growth rather than evidence of a literal carrying capacity of the research field.

**Fig. 2:**
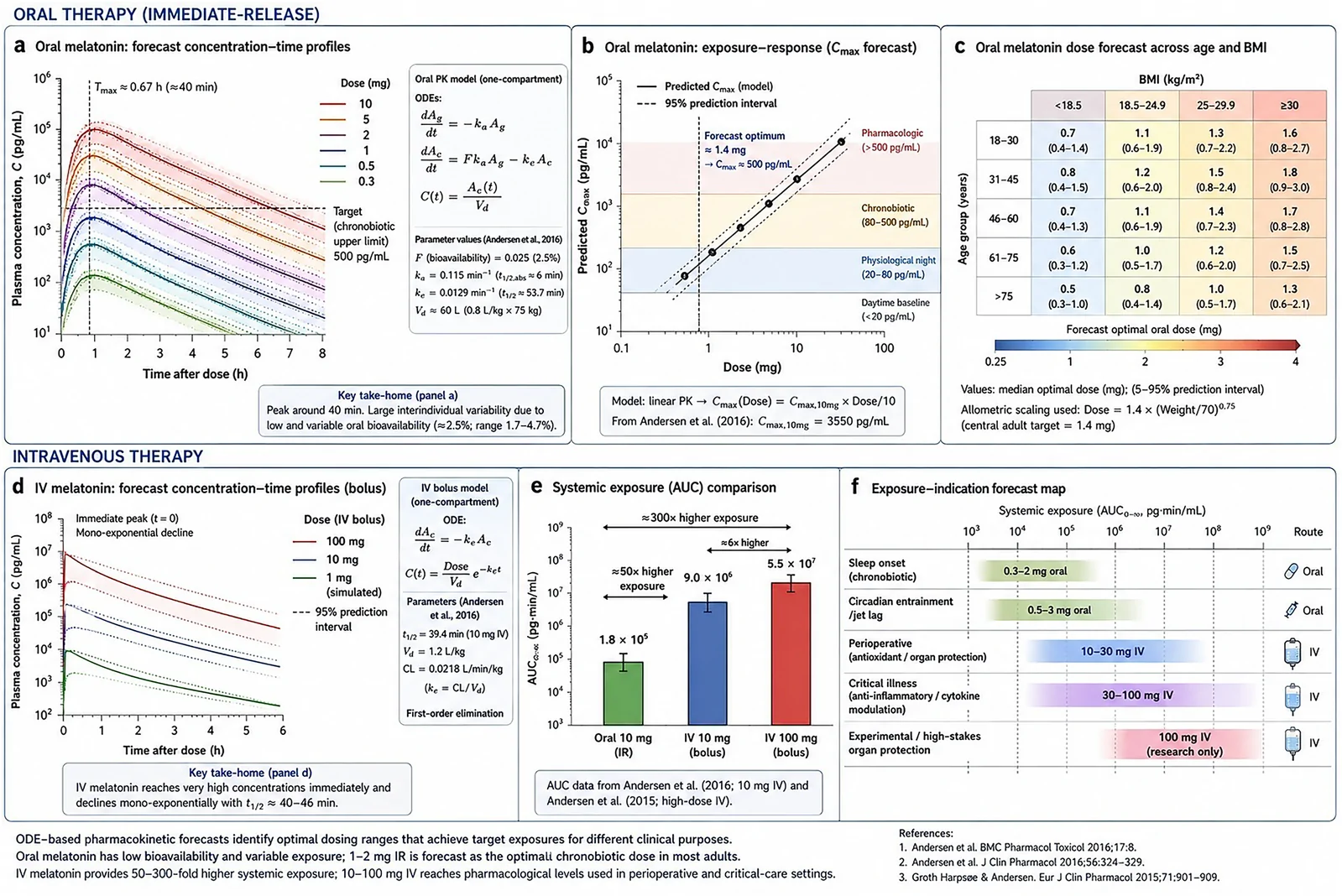
ODE-based pharmacokinetic modelling and dose-exposure forecasting for oral and intravenous melatonin. The figure presents a mathematical framework for comparing concentration-time profiles rather than prescribing treatment. Panel (a) illustrates simulated oral profiles across modelled doses, showing rapid absorption and exposure variability associated with low bioavailability. Panel (b) depicts the modelled dose-exposure relationship and an illustrative concentration target. Panel (c) shows exploratory body-size scaling; these estimates are not validated dosing recommendations. Panel (d) presents predicted IV bolus profiles, characterized by immediate peak exposure and first-order elimination. Panel (e) compares modelled systemic exposure between routes. Panel (f) maps exposure ranges to established or investigational contexts, distinguishing chronobiotic oral use from experimental high-exposure IV pharmacology. The IV ranges are theoretical pharmacokinetic forecasts and should not be interpreted as evidence of therapeutic superiority, validated clinical dosing, or practical implementation. Figures were prepared using Python (version 3.15; Python Software Foundation), including SciPy and Matplotlib, R (version 3.11.1; R Foundation for Statistical Computing), and Stata version 19 (StataCorp LLC)

Figure 2 summarizes the modelled exposure relationships and explicitly distinguishes pharmacokinetic forecasting from clinical recommendation.

The logistic and exponential models showed comparable support (ΔAICc < 2), providing no strong basis for preferring a saturating logistic trajectory over exponential growth (RMSE = X versus Y; AIC_c_ = X versus Y; ΔAIC_c_ for the exponential model = X). Residual inspection additionally showed greater systematic deviation from the exponential trajectory during the later observation period. The fitted logistic parameters were K=…, r=…, and t_0_=…. These results support the logistic model as the better descriptive model among the two candidate specifications tested; they should not be interpreted as demonstrating a true saturation limit for melatonin research.

## Insights on pharmacokinetics of oral and intravenous (IV) melatonin

### Oral melatonin

After oral administration, melatonin is generally absorbed rapidly, but its pharmacokinetic profile varies substantially across individuals and formulations. Immediate-release products usually reach peak plasma concentration within approximately 50 min, whereas prolonged-, delayed-, or sustained-release preparations alter the absorption phase [23, 31, 32]. The principal limitation of the oral route is low and variable absolute bioavailability because of extensive first-pass hepatic metabolism. A systematic review estimated average oral bioavailability at approximately 15%, but individual studies show a wide range [23].

In a crossover study of healthy volunteers receiving 10 mg oral melatonin, Andersen et al. reported a median bioavailability of approximately 3% and an elimination half-life of about 54 min [15]. The finding is important not because it defines a universal dose, but because it demonstrates that a fixed oral dose can generate limited and variable systemic exposure.

For chronobiotic use, formulation and timing are at least as important as nominal dose. Immediate-release preparations may be appropriate when a short exposure and phase-shifting signal are intended, whereas prolonged-release formulations extend exposure and may better approximate aspects of nocturnal secretion [31, 32]. The clinical effect also depends on circadian phase; therefore, dose-response relationships for sleep and circadian outcomes cannot be interpreted independently of administration time.

### Intravenous melatonin

Intravenous administration bypasses gastrointestinal absorption and first-pass metabolism, yielding complete systemic availability and a more predictable concentration-time profile. In healthy volunteers, 10 mg IV melatonin had an elimination half-life of about 39 min, a mean volume of distribution near 1.2 L/kg, and clearance of approximately 0.0218 L/min/kg [15]. These values establish rapid distribution and elimination but do not identify an optimal therapeutic indication or dose.

Earlier IV studies also described a rapid biexponential decline after administration [33, 34], while high-dose studies confirmed that substantially higher plasma concentrations can be achieved [22]. This makes IV administration useful as a pharmacological comparator and potentially as a research route for controlled exposure. Whether that exposure has practical clinical merit must be established separately for each indication through formulation, safety, PK-PD, efficacy, regulatory, and implementation studies.

Oral and IV melatonin should therefore be viewed as complementary pharmacokinetic models rather than competing treatment strategies. Oral administration has the stronger clinical evidence base, especially for sleep and circadian applications. IV administration offers controlled high exposure but remains comparatively underexplored and should currently be regarded as investigational outside established research or hospital protocols.

## Recent preclinical studies on melatonin

### In vitro studies

Table 1 shows in vitro and animal studies regarding melatonin. Recent in vitro studies from 2020 to 2026 examine melatonin across several mechanistic domains, including redox regulation, mitochondrial function, apoptosis, autophagy, ferroptosis, inflammatory signaling, and cancer-cell biology. In cancer models, pharmacological concentrations have been associated with reduced proliferation, migration, or survival through pathways including NF-kB/MAPK, FAK/PD-L1, oxidative metabolism, and endoplasmic-reticulum stress [35]. These observations establish mechanistic hypotheses but do not, by themselves, demonstrate that comparable exposures or effects occur clinically.

**Table 1:** Representative preclinical studies relevant to melatonin-sensitive pathways and translational interpretation Studies are grouped according to the domains discussed in the text. The table emphasizes experimental model, direction of effect, and translational relevance rather than implying uniform therapeutic efficacy. Human Parkinson’s disease trials are included because they directly illustrate the distinction between sleep-related benefit and disease-modifying efficacy

Reference	Domain / setting	Study model / design	Melatonin exposure / intervention	Main pathway or endpoint	Main finding	Translational interpretation
[44]	Cross-system antioxidant biology	Systematic review of animal studies	Melatonin across heterogeneous animal models and dosing regimens	Antioxidant enzymes; lipid-peroxidation and oxidative-stress markers	Across animal studies, melatonin was associated with modulation of antioxidant defenses and lipid-peroxidation indices.	Supports redox-sensitive biological activity, but heterogeneity in species, dose, tissue, and endpoints limits direct clinical dose extrapolation.
[45]	Neurology / Parkinsonian model	MPP+-based experimental Parkinson’s disease mouse model	Melatonin supplementation in the experimental model	HSP70; autophagy; dopaminergic injury	Melatonin attenuated MPP+-related injury through mechanisms involving heat-shock protein signaling and autophagy.	Provides mechanistic neuroprotective evidence in a specific model; it does not establish a general benefit in human Parkinson’s disease.
[46]	Neurology / Parkinsonian models	6-OHDA and MPTP experimental Parkinsonian models in rats	Slow-release melatonin implantation versus pinealectomy or constant-light suppression of endogenous melatonin	Motor impairment after dopaminergic injury	Exogenous melatonin exacerbated motor/behavioral impairment, whereas reduction of endogenous melatonin by pinealectomy or constant light reduced severity.	Demonstrates that melatonin effects can be disease- and context-dependent and may oppose generalized assumptions of neuroprotection.
[47]	Neurology / Parkinsonian models	Chronic bilateral 6-OHDA experimental model	Melatonin analogues ML-23 and S-20,928; receptor-antagonist activity considered	Motor and regulatory deficits after dopamine depletion	Selected melatonin analogues improved recovery; the authors proposed that melatonin-receptor antagonism may contribute to benefit.	Supports the need to consider receptor biology and direction of melatonin signaling rather than treating melatonin as uniformly beneficial.
[48]	Human Parkinson’s disease / sleep	Randomized, double-blind, placebo-controlled clinical study	Melatonin 3 mg orally, 1 h before bedtime for 4 weeks	Subjective sleep quality; polysomnography; motor dysfunction	Subjective sleep quality improved, whereas polysomnographic abnormalities and motor dysfunction did not improve.	Suggests symptom-specific sleep benefit without evidence of motor or disease-modifying efficacy.
[49]	Human Parkinson’s disease / REM-sleep behaviour disorder	Randomized, double-blind, placebo-controlled trial; *n* = 30	Prolonged-release melatonin 4 mg orally at bedtime for 8 weeks	RBD event frequency; tolerability	The primary RBD endpoint was not improved versus placebo; mild headache, fatigue, and morning sleepiness were reported.	Shows that even within Parkinson’s disease, efficacy depends on target symptom, formulation, timing, and endpoint.
[50]	Metabolic / cardiovascular stress	Diabetic rat model exposed to acute exhaustive exercise	Melatonin treatment in vivo	Cardiac oxidative stress and redox injury	Melatonin attenuated exercise-associated cardiac oxidative stress in diabetic rats.	Mechanistic support for redox modulation; clinical cardiovascular efficacy and transferable dosing remain unestablished.
[51]	Metabolic / diabetic heart	Systematic review and meta-analysis of rodent models	Melatonin, alone or with exercise training, across included animal studies	Cardiac function; oxidative stress; metabolic and inflammatory outcomes	Rodent studies reported improvements in several indices of diabetic cardiac injury and metabolic/redox dysfunction.	Aggregated animal evidence supports further study but cannot define a human therapeutic regimen.
[52]	Developmental cardio-kidney-metabolic biology	Review of pregnancy/lactation animal models complicated by oxidative stress	Maternal melatonin treatment across experimental models	Offspring cardiovascular, kidney, and metabolic outcomes	Melatonin was associated with modulation of developmental oxidative-stress pathways and later cardio-kidney-metabolic phenotypes.	Highlights developmental and timing-dependent effects; translation requires careful consideration of maternal-fetal exposure and long-term safety.
[53]	Metabolic / bone microenvironment	High-glucose experimental osteogenic model involving macrophage–BMSC crosstalk	Melatonin treatment in the experimental system	NRF2; autophagy; osteogenic differentiation	Melatonin promoted osteogenic differentiation under high-glucose stress through NRF2/autophagy-related signaling.	Identifies an exposure-sensitive metabolic-stress pathway but does not establish a clinical indication or dose.
[54]	Inflammation / sepsis	In vivo rat model of sepsis-induced liver injury	Melatonin treatment in vivo	Inflammatory mediators; oxidative injury; liver pathology	Melatonin reduced inflammatory and oxidative-injury indices in experimental sepsis-associated liver damage.	Supports anti-inflammatory pathway modulation in a specific animal model; human critical-care efficacy requires direct testing.
[55]	Pulmonary toxicology	Systematic review of in vivo alkylating-agent-induced pulmonary toxicity models	Melatonin across included animal studies	Oxidative injury; inflammation; pulmonary tissue pathology	Preclinical studies reported protective changes in pulmonary oxidative/inflammatory injury under selected conditions.	Species, toxicant, dose, and treatment-timing heterogeneity prevent direct extrapolation to clinical pulmonary disease.
[56]	Developmental / reproductive biology	Preclinical fetal-hypoxia model with ovarian granulosa-cell injury	Melatonin supplementation	Autophagy; mitochondrial function; ovarian injury	Melatonin mitigated fetal-hypoxia-associated autophagic and mitochondrial impairment in ovarian granulosa cells.	Provides a mechanistic developmental/reproductive signal whose clinical relevance depends on exposure, developmental stage, and safety.
[57]	Cancer / breast cancer	Systematic review of experimental breast-cancer models	Melatonin across heterogeneous dose-response regimens	Tumor growth; apoptosis; oncostatic mechanisms	Selected experimental models reported dose-related oncostatic and pro-apoptotic effects.	Tumor biology, model quality, timing, dose, and achievable human exposure vary substantially; findings are hypothesis-generating rather than practice-guiding.

A second recurring mechanistic domain is mitochondrial and cellular-stress regulation. Studies in reproductive cells and other cellular systems report reduced reactive oxygen species, preservation of mitochondrial function, modulation of apoptosis, and changes in SIRT1/PGC-1alpha, NRF2, GPX4, NAD+ metabolism, autophagy, and ferroptosis-related pathways [36–42]. The convergence of these pathways is biologically informative, but their translational relevance depends on concentration, exposure duration, cell type, and whether the required tissue concentrations are achievable in humans.

Reproductive-cell models illustrate this exposure problem particularly clearly. Experimental work has reported partial protection against toxicant- or metabolic-stress injury, with changes in ROS, mitochondrial integrity, DNA damage, and developmental competence [37, 41, 42]. Such findings should be interpreted as context-specific preclinical observations rather than as evidence for generalized supplementation or clinical use.

Overall, the in vitro literature supports a network of interconnected cellular effects rather than a single unified therapeutic mechanism. Many studies use high pharmacological concentrations, short exposure windows, or isolated systems. The key translational question is therefore not whether an effect can be demonstrated in vitro, but whether a clinically feasible regimen can reproduce the relevant exposure and target engagement without unacceptable off-target effects [43].

The translational relevance of these findings depends critically on exposure. Conventional oral administration may not reproduce concentrations used in many experimental systems, while IV administration can generate much higher systemic concentrations. However, the ability to reach a concentration does not establish benefit, safety, tissue-level exposure, or practical utility. The preclinical-clinical gap should therefore be framed as an unresolved PK-PD problem, not as an argument for preferential IV treatment.

### In vivo studies with laboratory animals

Recent animal studies provide evidence for melatonin-sensitive pathways in neurological, metabolic, inflammatory, reproductive, and cancer models, but the direction and clinical relevance of these effects are not uniform. A 2024 systematic review of animal studies reported changes in antioxidant enzymes and lipid-peroxidation markers [44]. In a mouse Parkinsonian model, melatonin reduced MPP+-related dopaminergic injury through mechanisms involving HSP70 and autophagy [45], whereas other experimental work has reported worsening of motor deficits with enhanced melatonin signaling and improvement after reduction or antagonism of melatonin activity [46, 47]. These apparently divergent findings illustrate why disease-specific biology must take precedence over generalized assumptions of neuroprotection.

The neurological literature is therefore especially instructive. In Parkinson’s disease, human trials have primarily examined sleep-related outcomes rather than disease-modifying effects. A small randomized trial found improved subjective sleep quality without improvement in motor dysfunction [48], and a randomized trial of prolonged-release melatonin for REM-sleep behaviour disorder did not improve the primary endpoint and reported mild headache, fatigue, or morning sleepiness [49]. These findings do not support indiscriminate use of melatonin in Parkinson’s disease and demonstrate that timing, target symptom, receptor biology, and disease context must be considered explicitly.

In metabolic and cardiovascular models, melatonin has mainly been evaluated against oxidative and metabolic stress. Reported improvements in redox markers, glucose homeostasis, inflammation, apoptosis, or tissue injury [50–53] provide mechanistic support for further study, but they do not establish a transferable dose or clinical indication.

Inflammation, sepsis, pulmonary injury, and developmental models similarly report changes in cytokines, NF-kB-related signaling, oxidative injury, or tissue pathology [54–56]. These effects are heterogeneous across species and experimental designs. Their value for this review lies in identifying candidate exposure-sensitive pathways that require confirmation in humans, not in supporting broad therapeutic claims.

Cancer models also remain heterogeneous. Experimental studies and systematic reviews have reported effects on tumor growth or apoptosis under selected conditions [57]. Dose, tumour biology, timing, model quality, and achievable exposure differ substantially, preventing direct extrapolation to clinical practice.

Taken together, the preclinical literature provides mechanistic hypotheses across several interconnected pathways but also exposes a recurrent translational limitation: the administered doses and achieved concentrations often do not map cleanly onto clinically used regimens. This mismatch provides the rationale for the route-specific PK analysis and the model-based exposure forecasts presented below.

## Recent clinical evidence and consistency across applications

Table 2 summarizes recent clinical studies using oral, sublingual, buccal, or topical-oral melatonin [58–94]. The studies demonstrate marked heterogeneity in indication, dose, formulation, treatment duration, and endpoint. Rather than supporting a uniform pleiotropic therapeutic effect, the human literature identifies different levels of evidence across domains. Sleep and circadian outcomes are most directly aligned with established melatonin physiology, whereas perioperative, analgesic, cardiovascular, renal, inflammatory, and neuroprotective applications show more variable efficacy. Most studies did not measure systemic melatonin exposure, limiting the ability to determine whether outcomes reflect pharmacodynamic differences, inadequate exposure, formulation-dependent bioavailability, or interindividual pharmacokinetic variability (Table 1).

**Table 2:** 2 A Clinical studies using oral, sublingual, buccal or topical-oral melatonin exposure (2020–2026)Included studies are human clinical studies published or indexed from 2020 to May 2026. Sublingual and oral-cavity gel/mouthwash are retained here because they are non-IV clinical exposure routes and are often discussed together with oral supplementation in perioperative/supportive-care settings

Reference	Population/setting	Design	Route and dose/regimen	Main endpoints	Main finding / relevance	Clinical domain	Source
[58]	Critically ill adult ICU patients; sleep disturbance	Randomized placebo-controlled clinical trial	Oral melatonin supplementation; dose not specified in retrieved abstract snippet	Sleep quality, ICU sleep measures, tolerability	Melatonin was associated with improved sleep quality; proposed for routine ICU care evaluation.	Sleep/ICU	PMID 33,048,904
[59]	Adults with bilateral multiple rib fractures receiving thoracic epidural analgesia	Prospective double-blind randomized controlled study; *n* = 80	Oral melatonin 5 mg about 1 h before epidural infusion, then every 12 h until bupivacaine stopped	Morphine consumption, bupivacaine infusion rate/duration, VAS pain	Reduced morphine use and bupivacaine requirement; prolonged analgesic effects vs. placebo.	Pain/trauma anesthesia	PMID 32,341,662; PMCID PMC7166071
[60]	Periodontitis patients undergoing nonsurgical periodontal therapy	Clinical trial of adjunctive oral melatonin after NSPT	Oral melatonin supplementation; exact dose not shown in retrieved snippet	Periodontal clinical response, host modulation	Oral melatonin was evaluated as a host-modulating adjunct to periodontal therapy.	Dentistry/periodontal inflammation	PMID 31,407,353
[61]	Preterm newborns	Clinical trial / protocol-oriented neonatal neuroprotection study	Oral melatonin; neonatal dosing evaluated in early extrauterine life	Neuroprotection feasibility, oxidative stress background, neonatal exposure	Study rationale: preterm infants cannot mount mature endogenous melatonin rhythm; oral melatonin proposed as neuroprotectant.	Neonatology/neuroprotection	PMID 33,482,894
[62]	Preterm newborns at risk of oxidative stress	Prospective randomized double-blind placebo-controlled pilot trial; *n* = 36	Oral melatonin 0.5 mg/kg once daily from day 1 of life	Plasma melatonin, NPBI, AOPP, F2-isoprostanes	Higher plasma melatonin and lower F2-isoprostanes at 48 h; no major differences in NPBI/AOPP.	Neonatology/oxidative stress	PMID 34,840,669; PMCID PMC8626170
[63]	Head and neck cancer patients at risk of oral mucositis	Randomized placebo-controlled phase II trial	High-dose melatonin oral gel mouthwash	Prevention/treatment of radiation/chemotherapy-associated oral mucositis	Evaluated topical/oral cavity melatonin gel for mucositis prevention and treatment.	Oncology/supportive care	PMID 33,738,704
[64]	Patients with heart failure with reduced ejection fraction (HFrEF)	Randomized clinical trial / MeHR-related cardiovascular study	Oral melatonin supplementation; dose not specified in retrieved snippet	Endothelial function; diabetes subgroup	May improve endothelial function in HFrEF; benefit less evident in diabetic patients.	Cardiovascular/HFrEF	PMID 34,184,295
[65]	Patients with HFrEF	Randomized clinical trial (MeHR trial)	Oral melatonin supplementation; dose not specified in retrieved snippet	NT-proBNP, quality of life, clinical status	Reduced NT-proBNP and improved quality of life; proposed as potentially beneficial supplement.	Cardiovascular/HFrEF	PMID 35,170,783
[66]	Hospitalized COVID-19 patients	Open-label randomized controlled trial	Oral melatonin adjunctive therapy; commonly reported as 3 mg three times daily in COVID-19 clinical discussions	Clinical recovery, symptoms, inflammatory/clinical outcomes	Improved clinical recovery signals in COVID-19; evidence remains limited and heterogeneous.	COVID-19/inflammation	PMID/PMCID: PMCID PMC9015545
[67]	Healthcare workers at high SARS-CoV-2 exposure risk	Multicenter randomized double-blind placebo-controlled prophylaxis trial; *n* = 308 treated	Oral melatonin 2 mg nightly for 12 weeks	SARS-CoV-2 infections, adverse events, somnolence	Did not prevent SARS-CoV-2 infection; more treatment-related somnolence; no severe treatment-related AEs.	COVID-19 prophylaxis	PMCID PMC8876218; NCT04353128
[68]	Patients with severe COVID-19 pneumonia	Randomized clinical trial	Oral melatonin plus standard care; exact regimen not shown in snippet	Ordinal clinical status, mechanical ventilation, discharge timing, safety	Improved clinical status and reduced IMV requirement signals with safe profile.	COVID-19/severe pneumonia	PMCID PMC9676876
[69]	Lumbar laminectomy/discectomy patients	Randomized clinical study	Preoperative oral melatonin; exact dose not shown in retrieved snippet	Postoperative pain severity and analgesic outcomes	Investigated oral melatonin as preoperative analgesic adjunct in spine surgery.	Perioperative pain	PMID 36,475,244
[70]	Patients undergoing elective hernia repair	Randomized placebo-controlled study	Oral preoperative melatonin; exact dose not shown in retrieved snippet	Preoperative anxiety	Did not reduce preoperative anxiety; baseline anxiety was low, limiting generalizability.	Perioperative anxiety	PMID 36,106,858
[71]	Women undergoing total abdominal hysterectomy	Double-blind randomized clinical trial	Single preoperative 5 mg prolonged-release oral capsule	Intraoperative midazolam use, OAA/S sedation, hospital stay	Reduced intraoperative midazolam consumption and hospital stay; sedative effect observed 30–90 min after dosing.	Perioperative sedation/anxiety	PMID 35,636,937
[72]	Migraine patients	Randomized placebo-controlled prophylaxis study	Oral melatonin; exact regimen not shown in retrieved snippet	Responder rate, migraine severity/frequency-related outcomes	Melatonin showed prophylactic benefit, including better responder rate vs. placebo.	Neurology/migraine	PMID 36,445,912
[73]	Patients undergoing cardiac surgery	Single-center double-blind randomized clinical trial	Oral melatonin 30 mg vs. placebo	Prevention of acute kidney injury	Assessed high-dose oral melatonin for AKI prevention in cardiac surgery.	Cardiac surgery/renal protection	PMID 36,586,514
[74]	Adults undergoing general anesthesia with intubation	Prospective double-blind randomized study; *n* = 50	Oral melatonin 6 mg 2 h before surgery	Hemodynamic response, propofol/isoflurane/fentanyl requirements	Reduced MAP before/after induction and reduced propofol and isoflurane requirements; did not fully attenuate intubation responses.	Anesthesia/premedication	PMID 38,269,180
[75]	Women undergoing induction of labor	Double-blind randomized placebo-controlled trial	Melatonin during induction of labor; route/dose not visible in retrieved snippet, likely oral supplementation	Cesarean birth requirement	Tested whether melatonin reduces need for cesarean birth during induction.	Obstetrics/reproductive medicine	PMID 39,446,608
[76]	Rotating night-shift workers	Double-blind randomized placebo-controlled study	Bedtime oral melatonin for 12 weeks	Insulin resistance, sleep quality	Improved sleep quality but did not significantly affect insulin resistance.	Metabolic/sleep/circadian	PMID 38,029,806
[77]	Mild cognitive impairment	Randomized controlled feasibility trial	25 mg melatonin for 3 months	Brain oxidative stress, sleep, feasibility, tolerability	Feasibility/tolerability study targeting brain oxidative stress and sleep in MCI.	Neurology/aging	PMID 39,702,983
[78]	Older hospitalized medical inpatients with delirium	Double-blind randomized placebo-controlled trial	Oral immediate-release melatonin 5 mg nightly for 5 nights	Delirium severity	Did not reduce delirium severity; useful negative clinical evidence.	Geriatrics/delirium	PMID 38,438,279
[79]	Patients with zygomaticomaxillary complex fractures	Randomized placebo-controlled study; *n* = 64	Oral melatonin prophylaxis for 15 consecutive days	Postoperative pain, sensory abnormalities/recovery	Evaluated postoperative pain and sensory recovery after facial fracture surgery.	Maxillofacial surgery/pain	PMID 38,609,755
[80]	Women with facial melasma	Double-blind randomized placebo-controlled clinical trial	Oral melatonin 5 mg	Melasma severity/treatment response	Assessed oral melatonin as antioxidant/photobiology-related adjunct in melasma.	Dermatology/melasma	PMID 38,226,560
[81]	Pediatric patients undergoing venipuncture	Randomized clinical study	Oral melatonin; exact dose not shown in retrieved snippet	Pain and anxiety during venipuncture	Investigated oral melatonin for procedural pain/anxiety reduction.	Pediatrics/procedural pain	PMID 39,359,816
[82]	Elderly postoperative patients	Multicenter randomized placebo-controlled pilot study	Oral melatonin 3 mg once preoperatively and for 7 days postoperatively	Postoperative delirium incidence; twice-daily delirium assessment	Pilot evidence for delirium prevention; requires larger confirmatory trials.	Geriatrics/perioperative delirium	PMID 39,998,812; PMCID PMC11856979
[83]	Iron-overloaded thalassemia with low bone mineral density	Randomized clinical supplementation trial	Oral melatonin 20 mg daily for 12 months	Lumbar spine/femoral neck BMD, safety	Alleviated lumbar spine BMD loss; long-duration skeletal endpoint study.	Bone/endocrine/hematology	PMID 40,329,508
[84]	Surgical patients; postoperative cognitive/delirium-related endpoint	Randomized trial of ketamine and melatonin	Oral melatonin 5 mg night before surgery plus perioperative ketamine/placebo design	Postoperative neurocognitive/delirium outcomes	Studied melatonin alone/combined with ketamine for postoperative neurocognitive protection.	Perioperative neuroprotection	PMID 40,846,975
[85]	Surgical/dental patients receiving preoperative anxiolysis	Randomized double-blind placebo-controlled clinical trial	Single preoperative 15 mg sublingual melatonin	Surgical discomfort, pain/anxiety-related outcomes	Evaluated sublingual high-dose melatonin; related report found no significant anxiety/pain benefit vs. placebo.	Perioperative/dental anxiolysis	PMID 41,243,001; PMID 41,578,909
[86]	Neonates undergoing venepuncture	Randomized placebo-controlled clinical study	Oral melatonin; exact regimen not shown in retrieved snippet	Procedural pain during venepuncture	Compared oral melatonin with placebo for neonatal venepuncture analgesia.	Neonatal procedural pain	PMID 40,900,317
[87]	Patients in a clinical QoL/sleep trial (disease not visible in snippet)	Randomized placebo-controlled study; *n* = 82	Oral melatonin 3 mg nightly for 6 weeks	Quality of life, sleep and associated outcomes	Nightly 3 mg oral melatonin evaluated for QoL and sleep improvements.	Sleep/QoL	PMID 40,790,573
[88]	Critically ill COVID-19 ICU patients	Quasi-experimental pragmatic clinical trial	Oral melatonin; dose range not shown in retrieved snippet	90-day mortality, ICU/hospital outcomes	Associated with lower 90-day mortality and improved ICU outcomes; nonrandomized/pragmatic design limits causal certainty.	Critical care/COVID-19	PMID 41,532,840; PMCID PMC12802531
[89]	Children with cerebral palsy and sleep disturbances	Randomized double-blind placebo-controlled trial	Oral melatonin 3 mg escalated to 10 mg vs. placebo for 12 weeks	Sleep Disturbance Scale and sleep outcomes	Assessed efficacy and safety of oral melatonin for sleep problems in cerebral palsy.	Pediatric neurology/sleep	PMID 41,369,870
[90]	Patients with advanced glaucoma and sleep disturbance	Randomized double-masked placebo-controlled crossover trial	Melatonin supplementation; exact dose not shown in retrieved snippet	Sleep quality and glaucoma-related sleep disruption	Investigated sleep quality in advanced glaucoma using crossover design.	Ophthalmology/sleep	PMID 41,386,535
[91]	Women with fibrocystic breast disease	Randomized double-blind placebo-controlled trial; *n* = 66	Melatonin supplementation; exact dose not shown in retrieved snippet	Pain, sleep quality, QoL	Reported improvements in pain, sleep quality and quality of life.	Pain/women’s health	PMCID PMC13067964
[92]	Hospitalized patients at delirium risk	Double-blind placebo-controlled trial of two doses	Oral melatonin; two-dose comparison	Delirium prevention	Evaluated melatonin doses for delirium prevention in hospitalized patients.	Geriatrics/delirium	PMCID PMC12853498
[93]	Primary progressive multiple sclerosis patients already receiving therapy	MELATOMS-1 safety/efficacy clinical study	Adjunctive high-dose oral melatonin; dose not shown in snippet	Hepatic safety and efficacy signals	Focused on hepatic safety of adjunctive high-dose melatonin in PPMS.	Neurology/MS	PMID 41,706,381
[94]	Chronic kidney disease patients	Clinical supplementation study	Melatonin supplementation; dose not shown in snippet	HDL-C, oxidative stress, sleep quality	Reported gentle HDL-C increase, oxidative stress decrease and sleep quality improvement.	Nephrology/metabolic/sleep	PMID 41,727,206

The most coherent clinical pattern concerns sleep and circadian regulation. Across ICU patients, night-shift workers, individuals with mild cognitive impairment, paediatric neurological disorders, glaucoma-associated sleep disturbance, and chronic disease populations, melatonin has been evaluated for sleep quality, timing, or circadian organization [58, 76, 77, 87–90]. Benefits were not uniform, but these indications are biologically and pharmacologically congruent with established chronobiotic actions. Importantly, chronobiotic efficacy depends on administration time relative to circadian phase; a dose cannot be judged independently of timing.

Perioperative and analgesic applications are less consistent. Trials have examined preoperative anxiety, anaesthetic sparing, postoperative pain, delirium prevention, procedural discomfort, and recovery [59, 69–71, 74, 79, 82, 84, 85]. Some report reductions in anaesthetic or analgesic requirements or improvement in selected outcomes, whereas others show little or no superiority over placebo. The heterogeneity argues against a single transferable mechanism and suggests that baseline risk, target symptom, timing, co-medication, treatment duration, and achieved exposure all require consideration.

Cardiovascular, renal, inflammatory, and critical-care studies show a similarly mixed pattern. Favourable changes have been reported in selected endothelial, biomarker, sleep, renal, and ICU outcomes [64–66, 73, 83, 87, 94], but designs range from pilot studies to pragmatic or quasi-experimental investigations, and dosing regimens are not directly comparable. Because critical illness, inflammation, altered hepatic perfusion, organ dysfunction, and polypharmacy can modify melatonin disposition, exposure should be measured rather than inferred from dose alone.

Negative and neutral findings are equally informative. Trials have failed to demonstrate consistent benefit for some delirium, anxiety, pain, and perioperative endpoints [70, 77, 85, 92]. Such results should not automatically be attributed to inadequate exposure; they may also indicate an unsuitable biological target, inappropriate timing, insufficient treatment duration, or genuine absence of clinically meaningful efficacy. This uncertainty is precisely why prospective PK-PD measurement is needed.

Taken together, Table 1 supports a hierarchy of evidentiary confidence rather than a generalized therapeutic claim. The evidence reviewed here is most consistent and physiologically aligned for sleep and circadian applications; non-circadian applications remain heterogeneous and often exploratory. The central unresolved issue is whether clinically achievable exposure is related to target engagement and outcome in a reproducible, indication-specific manner.(Table 3).

**Table 3:** 2B Clinical studies involving intravenous melatonin administration The IV literature is far smaller than the oral literature. To make the IV section usable, the table includes the key human IV pharmacokinetic/pharmacodynamic studies that predate 2020, plus 2020–2026 IV trial protocols identified in indexed sources. No large completed IV efficacy trial with published results was identified in the 2020-May 2026 search window

Reference	Population/setting	Design	Route and dose/regimen	Main endpoints	Main finding / relevance	Clinical domain	Source
[21]	Healthy male volunteers	Cohort crossover pharmacokinetic study	10 mg oral vs. 10 mg intravenous melatonin on separate study days; short IV infusion	Cmax, Tmax, absorption half-life, elimination half-life, AUC, oral bioavailability	Oral bioavailability about 3%; IV produced immediate high systemic exposure and enabled route comparison. Foundational IV PK reference, outside 2020–2026 window.	PK/route comparison	PMID 26,828,070 / PMCID PMC4759723 / NCT01923974
[109]	Healthy volunteers	Randomized double-blind placebo-controlled three-arm crossover experimental pain study	IV bolus 10 mg, IV bolus 100 mg, or placebo	Pain during contact burn injury, hyperalgesia area, inflammatory response	No meaningful analgesic, antihyperalgesic or peripheral anti-inflammatory effect of IV melatonin in acute burn injury model. Foundational negative IV PD study.	Experimental pain/pharmacodynamics	PMID 27,697,139
[31]	Healthy volunteers	Crossover high-dose IV pharmacokinetic and safety study	IV bolus 10 mg, IV bolus 100 mg and placebo	Plasma melatonin, half-life, Cmax, adverse effects, psychomotor testing	High-dose IV melatonin produced very high plasma concentrations with rapid elimination and no major objective psychomotor sedation in volunteers. Foundational IV safety/PK reference.	High-dose IV PK/safety	PMID 26,184,078
[110]	Adult ICU patients with COVID-19	Phase II single-center double-blind randomized placebo-controlled trial protocol	Intravenous melatonin (IVM); protocol designed to evaluate mortality in ICU COVID-19	Mortality, ICU clinical outcomes, safety	Published protocol; results not identified in indexed sources during this search. Important because it represents a true IV clinical trial design in critically ill adults.	Critical care/COVID-19	PMID 32,758,298; NCT04568863
[111]	Pediatric surgical patients	MELA-PAED randomized trial protocol	Intraoperative intravenous melatonin	Prevention of postoperative emergence agitation	Published protocol for IV intraoperative melatonin in children; outcome results not yet identified in indexed sources during this search.	Pediatric anesthesia/emergence agitation	PMID 37,904,610

## Model-based dose-exposure forecasting for oral and intravenous melatonin

### Oral melatonin

For immediate-release oral melatonin, a one-compartment model with first-order absorption and elimination was used as an exploratory representation of the concentration-time profile:

$$\:\frac{{dA}_{g}}{dt}=-{k}_{a}{A}_{g}$$

$$\:\frac{{dA}_{c}}{dt}=F{k}_{a}{A}_{g}-{k}_{e}{A}_{c}$$

$$\:C\left(t\right)=\frac{{A}_{c}\left(t\right)}{V}$$

where:

$$\:{A}_{g}\left(0\right)=D,\:{\:\:A}_{c}\left(0\right)=0$$

and closed-form solution:

$$\:C\left(t\right)=\frac{FD{k}_{a}}{V({k}_{a}-{k}_{e})}({e}^{-{k}_{e}t}-{e}^{-{k}_{a}t})$$

Model parameters were derived from published human pharmacokinetic data, principally the oral/IV crossover study by Andersen et al. [15] and supporting pharmacokinetic literature [23, 31, 32]. The model is used to examine exposure relationships and should not be interpreted as a validated dosing algorithm.

$$\:D=10\:\mathrm{m}\mathrm{g}$$

$$\:{C}_{max}=\mathrm{3,550}\:\mathrm{p}\mathrm{g}/\mathrm{m}\mathrm{l}$$

$$\:{T}_{max}=\:41\:\mathrm{m}\mathrm{i}\mathrm{n}=0.683\:\mathrm{h}$$

$$\:{t}_{1/2}=\:54\:\mathrm{m}\mathrm{i}\mathrm{n}\:=\:0.9\:\mathrm{h}$$

$$\:F=2.5\%=0.025$$

The elimination rate is:

$$\:{k}_{e}=\frac{\mathrm{l}\mathrm{n}2}{{t}_{1/2}}$$

$$\:{k}_{e}=\frac{0.693}{0.9}=0.770{\mathrm{h}}^{-1}$$

The absorption rate comes from:

$$\:{T}_{max}=\frac{ln\left({k}_{a}\right)-ln\left({k}_{e}\right)}{{k}_{a}-{k}_{e}}$$

Solving numerically:

$$\:{k}_{a}=2.484{h}^{-1}$$

The apparent volume comes from:

$$\:V=\frac{FD{k}_{a}}{{C}_{max}({k}_{a}-{k}_{e})}({e}^{-{k}_{e}{T}_{max}}-{e}^{{-k}_{a}{T}_{max}})$$

Applying the conversion:

$$\:\mathrm{3,550}\:\mathrm{p}\mathrm{g}/\mathrm{m}\mathrm{l}\:=0.00355\:\mathrm{m}\mathrm{g}/\mathrm{L}$$

we obtain the following result:

$$\:V\approx\:\:41.6\:\mathrm{L}$$

For exploratory dose-exposure scaling, linear pharmacokinetics were assumed within the modelled range:

$$\:{C_{\max }}\left( D \right) = {C_{\max \left( {10\:mg} \right)}}.\frac{D}{{10}}$$

A reference concentration was then used to illustrate how nominal dose maps onto predicted peak exposure:

$$\:{C}_{target}=500\:\mathrm{p}\mathrm{g}/\mathrm{m}\mathrm{l}$$

$$\:{D_{t\arg et}} = 10.\frac{{500}}{{3550}} = 1.41\:mg$$

This produces a model-derived immediate-release oral exposure estimate:

$$\:D\approx\:1.4\:\mathrm{m}\mathrm{g}$$

A conservative allometric approximation was additionally explored to illustrate the potential influence of body size on dose-exposure relationships:

$$\:{D}_{BMI}={D}_{70kg}(\frac{W}{70}{)}^{0.75}$$

For example, for a 90-kg subject the model yields:

$$\:D=1.4(\frac{90}{70}{)}^{0.75}\approx\:1.69\:\mathrm{m}\mathrm{g}$$

Because Andersen et al. did not provide robust BMI-stratified pharmacokinetic data, this calculation is exploratory and should not be interpreted as an assessed clinical recommendation [15].

The model was used to illustrate how a prespecified hypothetical concentration target maps onto oral dose under the selected pharmacokinetic assumptions. Because the target concentration was not derived from a validated clinical efficacy threshold, the resulting dose estimates should be interpreted solely as mathematical examples and not as optimal, recommended, or clinically validated doses.

For circadian applications, timing relative to biological phase remains an essential determinant that is not captured by this simple PK model.

### IV melatonin

For intravenous melatonin, the absorption compartment is absent and bioavailability is effectively F = 1. The resulting model is therefore simpler and is used here to illustrate the relationship between IV dose and systemic exposure, not to propose a preferred clinical regimen.

The ODE model for IV melatonin bolus is represented by:

$$\:\frac{{dA}_{c}}{dt}=-{k}_{e}{A}_{c}$$

$$\:C\left(t\right)=\frac{{R}_{0}}{CL}(1-{e}^{{-k}_{e}t})$$

At steady state:

$$\:{C}_{ss}=\frac{{R}_{0}}{CL}$$

So:

$$\:{R_0} = {C_{ss}}.CL$$

The clearance term was based on published human IV pharmacokinetic data [15, 22].

$$\:CL=0.0253\:L{min}^{-1}{Kg}^{-1}$$

For 70 kg:

$$\:CL = 0.0253.70 = 1.771\:L{\min ^{ - 1}}$$

Target:

$$\:{C}_{ss}=500\:pg{ml}^{-1}=0.0005\:mg{L}^{-1}$$

$$\:{R_0} = 0.0005.1.771$$

$$\:{R}_{0}=0.000886\:mg{min}^{-1}$$

Under the stated assumptions, the intravenous (IV) model was additionally used to illustrate the mathematical relationship between clearance, a prespecified concentration target, and the corresponding theoretical infusion rate. Because no validated therapeutic plasma concentration has been established for the non-circadian indications considered here, these calculations are presented solely as pharmacokinetic illustrations and should not be interpreted as clinical dosing guidance. Similarly, model-derived low-exposure bolus or infusion values are presented only to show the scale of dose required to reach selected concentration targets. They have not been prospectively validated for efficacy, safety, or practical implementation and should not be used as clinical dosing guidance.$\:{kg}^{-1}{h}^{-1}$

The 10- and 100-mg IV doses studied experimentally produce concentrations far above physiological nocturnal levels [22]. They should therefore be interpreted as high-exposure pharmacological experiments. Whether comparable exposure has practical merit for any proposed disorder requires indication-specific translational development, including formulation, infusion strategy, safety, target engagement, PK-PD analysis, randomized efficacy studies, regulatory evaluation, and implementation assessment.

Figure 2 summarizes the modelled exposure relationships and explicitly distinguishes pharmacokinetic forecasting from clinical recommendation.

## Discussion

This review integrates mechanistic evidence, clinical studies, and route-dependent pharmacokinetics within an exposure-based framework. Its principal conclusion is not that melatonin’s biological breadth supports broad therapeutic use, but that nominal dose alone is an inadequate descriptor of melatonin pharmacology. Biological target, timing, formulation, route, achieved systemic exposure, and patient context must be considered together.

The most important translational gap is the disconnect between experimental mechanisms and the exposures achieved in clinical practice. Many preclinical effects are reported at concentrations that may not be reproduced by conventional oral dosing, whereas most clinical trials administer a fixed dose without measuring plasma exposure. This prevents a direct test of whether the proposed mechanism was engaged in patients and limits interpretation of both positive and negative trials.

The oral-IV comparison should therefore be interpreted as an exposure contrast rather than a therapeutic hierarchy. Oral administration has the stronger evidence base for established chronobiotic indications. IV administration provides a means of achieving controlled high systemic exposure, but its clinical relevance remains to be demonstrated empirically and separately for each proposed indication.

The oral route illustrates why exposure cannot be inferred reliably from dose. In the Andersen crossover study, 10 mg oral melatonin produced a median peak concentration of approximately 3,550 pg/mL, a peak near 41 min, an elimination half-life near 54 min, and absolute bioavailability of only about 2.5-3% [15]. Across the broader pharmacokinetic literature, Cmax, AUC, clearance, volume of distribution, and bioavailability vary substantially [23]. Age, caffeine, smoking, feeding status, drug interactions, critical illness, and other patient factors further modify exposure [17–21, 23].

For chronobiotic applications, pharmacokinetics must also be integrated with circadian pharmacodynamics. A concentration achieved at an inappropriate biological time may not reproduce the desired phase-shifting or sleep effect. Thus, even a precise exposure estimate is insufficient unless administration timing is linked to the intended circadian target. This is a central example of why pleiotropy does not support ubiquitous dosing: different biological objectives require different temporal and exposure conditions [95–104].

IV administration produces a different concentration-time profile. Healthy-volunteer studies demonstrate immediate high exposure, rapid distribution, and relatively short elimination after 10–100 mg IV melatonin [15, 22]. These studies are useful for defining pharmacokinetic boundaries, but short-term tolerability in healthy volunteers does not establish safety or benefit in older adults, children, critically ill patients, or individuals with neurological disease. The practical merit of IV melatonin therefore remains an empirical question rather than an inference from bioavailability.

The ODE models should be read in this context. For oral immediate-release melatonin, the model reproduces a short-lived concentration profile and allows dose-exposure relationships to be explored under explicit assumptions. The approximately 1.4-mg estimate and the 0.5-2.0-mg illustrative range are not proposed as universal optimal doses; they are examples of how a chosen concentration target maps onto dose in a simplified model. They do not incorporate circadian phase, formulation-specific release, disease-related PK changes, or individual pharmacodynamics.

The IV model is even more explicitly hypothesis-generating. It shows how removing the absorption and first-pass components changes exposure and allows theoretical infusion or bolus targets to be calculated. It does not demonstrate that those targets are clinically desirable. Translation would require indication-specific dose-escalation, formulation, safety, tissue-exposure, target-engagement, efficacy, and health-system implementation studies, a programme that lies beyond the scope of this review.

The clinical evidence is consistent with this cautious interpretation. Sleep and circadian outcomes are the most pharmacologically coherent and the best supported by current human data, whereas perioperative, analgesic, cardiovascular, renal, inflammatory, neuroprotective, and critical-care applications show variable or inconclusive results. Positive findings in one setting should not be extrapolated to another simply because melatonin can influence multiple pathways.

Perioperative studies provide a clear example. Melatonin has been investigated as an anxiolytic, sedative-sparing agent, analgesic adjunct, and delirium-prevention strategy, yet outcomes are inconsistent. Rather than invoking pleiotropy to reconcile these findings, the more appropriate interpretation is that the target, timing, comparator, patient risk, concomitant treatment, and exposure differ among trials. Future studies should prospectively specify which mechanism is being tested and measure whether the intended exposure and target engagement are achieved.

Neurological applications require particular caution. Experimental studies can support opposite interpretations depending on model and endpoint. Parkinson’s disease is illustrative: some preclinical studies report neuroprotective effects of melatonin, whereas other experimental work reports worsening with melatonin signaling and improvement after melatonin reduction or receptor antagonism [45–47]. Human studies have mainly addressed sleep-related symptoms and have not established a disease-modifying motor benefit [48, 49]. Accordingly, generalized claims of neurological protection are not justified, and disease-specific adverse, neutral, and beneficial effects must be evaluated separately.

The principal contribution of the present modelling is therefore conceptual: it shifts interpretation from milligrams administered to exposure achieved. Cmax, AUC, half-life, clearance, formulation, route, and timing provide a more informative framework for generating testable PK-PD hypotheses. The same nominal dose may produce different exposures, and similar exposures may have different effects when delivered at different circadian phases or in different disease states.

Several uncertainties remain. Most available PK studies are small and involve healthy volunteers, often limiting generalizability to older adults, women, children, neonates, critically ill patients, and people with hepatic, renal, inflammatory, metabolic, or neurological disease. BMI- and age-specific forecasts are exploratory; exposure-response relationships for most non-sleep indications are undefined; clinical endpoints are heterogeneous; and long-term safety data for repeated high pharmacological exposure are limited.

Future research should therefore use prospective PK-PD designs. Studies should measure melatonin concentrations, predefine the target biological effect, document administration timing relative to circadian phase where relevant, characterize formulation and route, and assess target engagement before attributing clinical outcomes to a proposed mechanism. IV studies, if pursued, should begin with indication-specific translational questions rather than broad therapeutic claims and should include feasibility and implementation considerations alongside efficacy and safety.

Overall, the available evidence supports a restrained interpretation: oral melatonin has the clearest role in sleep and circadian applications, while non-circadian pharmacological uses remain indication-specific and variably supported. IV melatonin is best regarded at present as an investigational route for controlled exposure rather than as a superior or broadly applicable therapy.

### Limitations of the study

Several limitations should be considered when interpreting this review. The literature assessment was not conducted as a formal systematic review or meta-analysis, and the 1,076-record PubMed dataset was used for exploratory scientometric and narrative purposes rather than exhaustive evidence capture. Publication, language, database, and selection biases therefore remain possible.

No formal risk-of-bias assessment or certainty-of-evidence grading was performed for the included clinical studies; consequently, differences in study quality were not quantitatively incorporated into the synthesis.

The clinical literature is highly heterogeneous in design, population, dose, timing, formulation, route, treatment duration, and endpoint. This heterogeneity limits direct comparison and prevents a single quantitative estimate of efficacy across indications. Conclusions should therefore be understood as domain-specific patterns of evidence, not as proof of a unified pleiotropic therapeutic effect.

The pharmacokinetic evidence is also limited. Much of it derives from small studies in healthy volunteers, and melatonin disposition may differ with age, circadian phase, smoking, caffeine, oral contraceptive use, hepatic function, inflammation, critical illness, and concomitant medication. Extrapolation of model parameters to broader patient groups should therefore be undertaken cautiously.

Parameter uncertainty and between-subject pharmacokinetic variability were not propagated through the simulations to generate prediction intervals; consequently, the numerical outputs illustrate expected relationships under selected parameter values rather than the range of exposures expected in individual patients.

The mathematical models are deliberately simplified. The oral and IV models assume linear pharmacokinetics and first-order processes and do not capture complex distribution, formulation-specific release, nonlinear behaviour, tissue concentrations, circadian pharmacodynamics, disease-specific kinetics, or receptor-level target engagement. The scientometric models are likewise descriptive conceptual tools rather than precise forecasts.

The dose-exposure forecasts are exploratory theoretical estimates, not clinical recommendations. They do not incorporate the full range of interindividual variability, genetic factors, disease-specific targets, or clinically validated exposure-response thresholds. In particular, the IV calculations establish only what exposure might be predicted under stated assumptions; they do not establish therapeutic merit, feasibility, or a place in existing health systems.

A final limitation is the incomplete integration of PK and PD in the published clinical literature itself. Most trials report administered dose without measuring circulating melatonin or target engagement. As a result, the present review can identify exposure-related hypotheses but cannot retrospectively determine whether a negative study failed because of inadequate exposure or because the proposed mechanism was not clinically relevant. Prospective PK-PD studies are required to resolve this uncertainty.

## Conclusions

Melatonin has an established role in circadian and sleep-related physiology, but biological pleiotropy should not be equated with broad therapeutic efficacy. Evidence for non-circadian applications remains heterogeneous and should be evaluated separately for each indication, population, formulation, route, timing, and endpoint. Oral and intravenous administration produce substantially different pharmacokinetic profiles; these differences establish differences in exposure, not comparative therapeutic efficacy. Oral melatonin has the substantially larger clinical evidence base, whereas IV melatonin remains investigational.

The ODE models presented here are exploratory pharmacokinetic tools rather than validated clinical models. Their numerical outputs depend on simplifying assumptions and selected parameter values and should not be interpreted as recommended doses, therapeutic concentration targets, or evidence of clinical benefit. Their principal value is hypothesis generation: they illustrate why administered dose alone may inadequately characterize melatonin pharmacology. Prospective studies integrating dose, formulation, timing, route, measured systemic and, where feasible, tissue exposure, target engagement, safety, and clinically meaningful outcomes are required before exposure-based therapeutic strategies can be established.

Conceptualization: SC, SP; Methodology: SP, FMP; Software: SC; Validation: SC, GB, MM; Formal analysis: MM, GB; Investigation: SP, CG; Resources: MM; Data Curation: CG, FMP; Writing - Original Draft SC; Writing - Review & Editing. SC, SP; Visualization: SC; Supervision: SP, FMP; Project administration. SP; Funding acquisition: None.

## Data Availability

No datasets were generated or analysed during the current study.

## References

[1] Borjigin J, Zhang LS, Calinescu AA (2012) Circadian regulation of pineal gland rhythmicity. Mol Cell Endocrinol 349(1):13–19. 10.1016/j.mce.2011.07.009 21782887 PMC3202635

[2] Tordjman S, Chokron S, Delorme R, Charrier A, Bellissant E, Jaafari N, Fougerou C (2017) Melatonin: Pharmacology, Functions and Therapeutic Benefits. Curr Neuropharmacol 15(3):434–443. 10.2174/1570159X14666161228122115 28503116 PMC5405617

[3] Acuña-Castroviejo D, Escames G, Venegas C, Díaz-Casado ME, Lima-Cabello E, López LC, Rosales-Corral S, Tan DX, Reiter RJ (2014) Extrapineal melatonin: sources, regulation, and potential functions. Cell Mol Life Sci 71(16):2997–3025. 10.1007/s00018-014-1579-2 24554058 PMC11113552

[4] Chitimus DM, Popescu MR, Voiculescu SE, Panaitescu AM, Pavel B, Zagrean L, Zagrean AM (2020) Melatonin’s Impact on Antioxidative and Anti-Inflammatory Reprogramming in Homeostasis and Disease. Biomolecules 10(9):1211. 10.3390/biom10091211 32825327 PMC7563541

[5] Nabavi SM, Nabavi SF, Sureda A, Xiao J, Dehpour AR, Shirooie S, Silva AS, Baldi A, Khan H, Daglia M (2019) Anti-inflammatory effects of Melatonin: A mechanistic review. Crit Rev Food Sci Nutr 59(sup1):S4–S16. 10.1080/10408398.2018.1487927 29902071

[6] Slominski RM, Reiter RJ, Schlabritz-Loutsevitch N, Ostrom RS, Slominski AT (2012) Melatonin membrane receptors in peripheral tissues: distribution and functions. Mol Cell Endocrinol 351(2):152–166. 10.1016/j.mce.2012.01.004 22245784 PMC3288509

[7] Conti A, Conconi S, Hertens E, Skwarlo-Sonta K, Markowska M, Maestroni JM (2000) Evidence for melatonin synthesis in mouse and human bone marrow cells. J Pineal Res 28(4):193–202. 10.1034/j.1600-079x.2000.280401.x 10831154

[8] Carrillo-Vico A, Calvo JR, Abreu P, Lardone PJ, García-Mauriño S, Reiter RJ, Guerrero JM (2004) Evidence of melatonin synthesis by human lymphocytes and its physiological significance: possible role as intracrine, autocrine, and/or paracrine substance. FASEB J 18(3):537–539. 10.1096/fj.03-0694fje 14715696

[9] Mellor K, Papaioannou D, Thomason A, Bolt R, Evans C, Wilson M, Deery C (2022) Melatonin for pre-medication in children: a systematic review. BMC Pediatr 22(1):107. 10.1186/s12887-022-03149-w 35209863 PMC8876113

[10] Gao Y, Chen X, Zhou Q, Song J, Zhang X, Sun Y, Yu M, Li Y (2022) Effects of Melatonin Treatment on Perioperative Sleep Quality: A Systematic Review and Meta-Analysis with Trial Sequential Analysis of Randomized Controlled Trials. Nat Sci Sleep 14:1721–1736. 10.2147/NSS.S381918 36187327 PMC9519126

[11] Cho JH, Bhutani S, Kim CH, Irwin MR (2021) Anti-inflammatory effects of melatonin: A systematic review and meta-analysis of clinical trials. Brain Behav Immun 93:245–253. 10.1016/j.bbi.2021.01.034 33581247 PMC7979486

[12] Tobeiha M, Jafari A, Fadaei S, Mirazimi SMA, Dashti F, Amiri A, Khan H, Asemi Z, Reiter RJ, Hamblin MR, Mirzaei H (2022) Evidence for the Benefits of Melatonin in Cardiovascular Disease. Front Cardiovasc Med 9:888319. 10.3389/fcvm.2022.888319 35795371 PMC9251346

[13] Hsu CN, Tain YL (2026) Melatonin as a Redox Modulator in Developmental Programming: Implications for Cardiovascular-Kidney-Metabolic Risk. Int J Mol Sci 27(5):2390. 10.3390/ijms27052390 41828607 PMC12985806

[14] Menczel Schrire Z, Phillips CL, Chapman JL, Duffy SL, Wong G, D’Rozario AL, Comas M, Raisin I, Saini B, Gordon CJ, McKinnon AC, Naismith SL, Marshall NS, Grunstein RR, Hoyos CM (2022) Safety of higher doses of melatonin in adults: A systematic review and meta-analysis. J Pineal Res 72(2):e12782. 10.1111/jpi.12782 34923676

[15] Andersen LP, Werner MU, Rosenkilde MM, Harpsøe NG, Fuglsang H, Rosenberg J, Gögenur I (2016) Pharmacokinetics of oral and intravenous melatonin in healthy volunteers. BMC Pharmacol Toxicol 17:8. 10.1186/s40360-016-0052-2 26893170 PMC4759723

[16] Gooneratne NS, Edwards AY, Zhou C, Cuellar N, Grandner MA, Barrett JS (2012) Melatonin pharmacokinetics following two different oral surge-sustained release doses in older adults. J Pineal Res 52(4):437–445. 10.1111/j.1600-079X.2011.00958.x 22348451 PMC3682489

[17] Härtter S, Nordmark A, Rose DM, Bertilsson L, Tybring G, Laine K (2003) Effects of caffeine intake on the pharmacokinetics of melatonin, a probe drug for CYP1A2 activity. Br J Clin Pharmacol 56(6):679–682. 10.1046/j.1365-2125.2003.01933.x 14616429 PMC1884289

[18] Mistraletti G, Sabbatini G, Taverna M, Figini MA, Umbrello M, Magni P, Ruscica M, Dozio E, Esposti R, DeMartini G, Fraschini F, Rezzani R, Reiter RJ, Iapichino G (2010) Pharmacokinetics of orally administered melatonin in critically ill patients. J Pineal Res 48(2):142–147. 10.1111/j.1600-079X.2009.00737.x 20070489

[19] Papagiannidou E, Skene DJ, Ioannides C (2014) Potential drug interactions with melatonin. Physiol Behav 131:17–24. 10.1016/j.physbeh.2014.04.016 24732412

[20] Lenoir C, Rollason V, Desmeules JA, Samer CF (2021) Influence of Inflammation on Cytochromes P450 Activity in Adults: A Systematic Review of the Literature. Front Pharmacol 12:733935. 10.3389/fphar.2021.733935 34867341 PMC8637893

[21] Ewoldt TM, Abdulla A, Hunfeld N, Li L, Smeets TJL, Gommers D, Koch BCP, Endeman H (2022) The impact of sepsis on hepatic drug metabolism in critically ill patients: a narrative review. Expert Opin Drug Metab Toxicol 18(6):413–421. 10.1080/17425255.2022.2106215 35912845

[22] Andersen LP, Werner MU, Rosenkilde MM, Fenger AQ, Petersen MC, Rosenberg J, Gögenur I (2016) Pharmacokinetics of high-dose intravenous melatonin in humans. J Clin Pharmacol 56(3):324–329. 10.1002/jcph.592 26184078

[23] Harpsøe NG, Andersen LP, Gögenur I, Rosenberg J (2015) Clinical pharmacokinetics of melatonin: a systematic review. Eur J Clin Pharmacol 71(8):901–909. 10.1007/s00228-015-1873-4 26008214

[24] Sumsuzzman DM, Choi J, Jin Y, Hong Y (2021) Neurocognitive effects of melatonin treatment in healthy adults and individuals with Alzheimer’s disease and insomnia: A systematic review and meta-analysis of randomized controlled trials. Neurosci Biobehav Rev 127:459–473. 10.1016/j.neubiorev.2021.04.034 33957167

[25] Xie S, Fan W, He H, Huang F (2020) Role of Melatonin in the Regulation of Pain. J Pain Res 13:331–343. 10.2147/JPR.S228577 32104055 PMC7012243

[26] Perrone S, Cannavò L, Gerevini S, Buttarelli L, Leuratti P, Caselli E, Petrolini C, Raitano V, Beretta V, Carloni S From Oxidative Stress to Pain Modulation With Melatonin in Neonatal and Pediatric Care. Clin Ther 2026 May 16:S0149-2918(26)00134-7. doi: 10.1016/j.clinthera.2026.04.017 10.1016/j.clinthera.2026.04.017 42142977

[27] Lin JJ, Lin Y, Zhao TZ, Zhang CK, Zhang T, Chen XL, Ding JQ, Chang T, Zhang Z, Sun C, Zhao DD, Zhu JL, Li ZY, Li JL (2017) Melatonin Suppresses Neuropathic Pain via MT2-Dependent and -Independent Pathways in Dorsal Root Ganglia Neurons of Mice. Theranostics 7(7):2015–2032. 10.7150/thno.19500 28656058 PMC5485420

[28] Lopez-Canul M, Palazzo E, Dominguez-Lopez S, Luongo L, Lacoste B, Comai S, Angeloni D, Fraschini F, Boccella S, Spadoni G, Bedini A, Tarzia G, Maione S, Granados-Soto V, Gobbi G (2015) Selective melatonin MT2 receptor ligands relieve neuropathic pain through modulation of brainstem descending antinociceptive pathways. Pain 156(2):305–317. 10.1097/01.j.pain.0000460311.71572.5f 25599452

[29] Oh SN, Myung SK, Jho HJ (2020) Analgesic Efficacy of Melatonin: A Meta-Analysis of Randomized, Double-Blind, Placebo-Controlled Trials. J Clin Med 9(5):1553. 10.3390/jcm9051553 32455582 PMC7291209

[30] Andersen LPH, Gögenur I, Fenger AQ, Petersen MC, Rosenberg J, Werner MU (2015) Analgesic and antihyperalgesic effects of melatonin in a human inflammatory pain model: a randomized, double-blind, placebo-controlled, three-arm crossover study. Pain 156(11):2286–2294. 10.1097/j.pain.0000000000000284 26164585

[31] Ait Abdellah S, Raverot V, Gal C, Guinobert I, Bardot V, Blondeau C, Claustrat B (2023) Bioavailability of Melatonin after Administration of an Oral Prolonged-Release Tablet and an Immediate-Release Sublingual Spray in Healthy Male Volunteers. Drugs R D 23(3):257–265. 10.1007/s40268-023-00431-9 37438493 PMC10439092

[32] Moroni I, Garcia-Bennett A, Chapman J, Grunstein RR, Gordon CJ, Comas M (2021) Pharmacokinetics of exogenous melatonin in relation to formulation, and effects on sleep: A systematic review. Sleep Med Rev 57:101431. 10.1016/j.smrv.2021.101431 33549911

[33] Mallo C, Zaĭdan R, Galy G, Vermeulen E, Brun J, Chazot G, Claustrat B (1990) Pharmacokinetics of melatonin in man after intravenous infusion and bolus injection. Eur J Clin Pharmacol 38(3):297–301. 10.1007/BF00315035 2340850

[34] Andersen LW, Holmberg MJ, Granfeldt A (2025) Melatonin for all? Concerns about recent guidelines. J Crit Care 90:155193. 10.1016/j.jcrc.2025.155193 40701057

[35] Wu CC, Yang PF, Chang SJ, Pan MR, Li CL, Wu CC, Kan JY, Chen FM, Hou MF, Luo CW (2026) Melatonin inhibits FAK signaling to suppress PD-L1 expression and enhance chemosensitivity in triple-negative breast cancer. Int J Med Sci 23(3):876–888. 10.7150/ijms.127669 41799764 PMC12964572

[36] Zhang L, Liu K, Liu Z, Tao H, Fu X, Hou J, Jia G, Hou Y (2024) In pre-clinical study fetal hypoxia caused autophagy and mitochondrial impairment in ovary granulosa cells mitigated by melatonin supplement. J Adv Res 64:15–30. 10.1016/j.jare.2023.11.008 37956860 PMC11464463

[37] Wu F, Qu H, Zhang Y, Huang F, Niu P, Chen H, Fang D, Gao Q (2025) Melatonin enhances reproductive capability in HT-2 toxin-treated oocytes via mitochondrial restoration. Ecotoxicol Environ Saf 303:119038. 10.1016/j.ecoenv.2025.119038 40945088

[38] Zhang J, Zhu L, Zhou J, Yu Q, Yang G, Luo C, Meng J, Mao K, Liu J, Mou D, Yang X (2026) Melatonin improves osteogenic differentiation in a high-glucose environment by activating NRF2 to promote autophagy through the regulation of cross-talk between macrophages and bone marrow mesenchymal stem cells. Inflamm Res 75(1):112. 10.1007/s00011-026-02267-w 42096091

[39] Kang JY, Xu MM, Chen TT, Wang MY, Wang QY, Tan YY, Wei YY, Fei GH (2026) Melatonin inhibits ferroptosis by activating CREB-GPX4/ferritin axis in cigarette smoke-induced chronic obstructive pulmonary disease. Int Immunopharmacol 173:116314. 10.1016/j.intimp.2026.116314 41643263

[40] Lan T, Yang W, Yan B, Guo W, Zhang Y (2025) Melatonin attenuates intervertebral disc degeneration by restoring mitochondrial homeostasis through PGC-1α signaling pathway. Cell Mol Life Sci 82(1):330. 10.1007/s00018-025-05877-5 40886189 PMC12399489

[41] Yang G, Fang J, Wang H, Kong Y, Yang Q, Zhai J, Wang C, Jiang R, Sun Y, Yao G (2026) Melatonin promotes embryonic development by enhancing tryptophan metabolism and NAD + synthesis. BMC Biol 24(1):73. 10.1186/s12915-026-02549-z 41692734 PMC13014840

[42] Zhang K, Guo J, Wang S, Min C, Wang J, Liu H, Fang Y, Ding H, Zhao J, Ma X, Lu W (2025) Melatonin protects bovine oocyte from βHB-induced oxidative stress through the Nrf2 pathway. Theriogenology 2 34:64–72. 10.1016/j.theriogenology.2024.11.025 39644523

[43] Monteiro KKAC, Shiroma ME, Damous LL, Simões MJ, Simões RDS, Cipolla-Neto J, Baracat EC, Soares-Jr JM (2024) Antioxidant Actions of Melatonin: A Systematic Review of Animal Studies. Antioxid (Basel) 13(4):439. 10.3390/antiox13040439 PMC11047453 38671887

[44] Guo Y, Liu C (2025) Melatonin attenuates MPP^+^-induced autophagy *via* heat shock protein in the Parkinson’s disease mouse model. PeerJ 13:e18788. 10.7717/peerj.18788 39866567 PMC11758912

[45] Li W, Chen Q, Fu H (2025) The role of melatonin in affecting cognitive dysfunction in acute sleep deprivation mice through the nuclear factor kappaB pathway and oxidative stress. Transl Neurosci 16(1):20250379. 10.1515/tnsci-2025-0379 41079404 PMC12514685

[46] Willis GL, Armstrong SM (1999) A therapeutic role for melatonin antagonism in experimental models of Parkinson’s disease. Physiol Behav 66(5):785–795. 10.1016/S0031-9384(99)00023-2 10405106

[47] Willis GL, Robertson AD (2004) Recovery of experimental Parkinson’s disease with the melatonin analogues ML-23 and S-20928 in a chronic, bilateral 6-OHDA model: a new mechanism involving antagonism of the melatonin receptor. Pharmacol Biochem Behav 79(3):413–429. 10.1016/j.pbb.2004.08.011 15582013

[48] Medeiros CAM, de Bruin PFC, Lopes LA, Magalhaes MC, de Lourdes Seabra M, de Bruin VMS (2007) Effect of exogenous melatonin on sleep and motor dysfunction in Parkinson’s disease. A randomized, double blind, placebo-controlled study. J Neurol 254(4):459–464. 10.1007/s00415-006-0390-x 17404779

[49] Gilat M, Jackson AC, Marshall NS, Hammond D, Mullins AE, Hall JM, Fang BAM, Yee BJ, Wong KKH, Grunstein RR, Lewis SJG (2020) Melatonin for rapid eye movement sleep behavior disorder in Parkinson’s disease: a randomised controlled trial. Mov Disord 35(2):344–349. 10.1002/mds.27886 31674060 PMC7027846

[50] Kim P, Garner N, Tatkovic A, Parsons R, Chunduri P, Vukovic J, Piper M, Pfeffer M, Weiergräber M, Oster H, Rawashdeh O (2024) Melatonin’s role in the timing of sleep onset is conserved in nocturnal mice. NPJ Biol Timing Sleep 1(1):13. 10.1038/s44323-024-00013-1 39493889 PMC11530376

[51] Nguyen C, Figueiroa RI, da Silva CM, Hatanaka E, Sweeney G, Lambertucci RH (2025) Melatonin attenuates cardiac oxidative stress in diabetic rats following acute exhaustive exercise. Cell Stress Chaperones 30(6):100126. 10.1016/j.cstres.2025.100126 41110543 PMC12616075

[52] Rahbarghazi A, Alamdari KA, Rahbarghazi R, Salehi-Pourmehr H (2023) Co-administration of exercise training and melatonin on the function of diabetic heart tissue: a systematic review and meta-analysis of rodent models. Diabetol Metab Syndr 15(1):67. 10.1186/s13098-023-01045-6 37005639 PMC10067225

[53] Benedeto-Stojanov D, Ničković VP, Petrović G, Rancić A, Grgov I, Nikolić GR, Marčetić ZP, Popović MR, Lazarević M, Mitić KV, Sokolović D (2023) Melatonin as a Promising Anti-Inflammatory Agent in an In Vivo Animal Model of Sepsis-Induced Rat Liver Damage. Int J Mol Sci 25(1):455. 10.3390/ijms25010455 38203627 PMC10779228

[54] Sola E, Morales-García JA, López-Muñoz F, Ramos E, Romero A (2025) Vivo Evidence of Melatonin’s Protective Role in Alkylating-Agent-Induced Pulmonary Toxicity: A Systematic Review. Antioxid (Basel) 14(6):712. 10.3390/antiox14060712 PMC12189487 40563344

[55] Tain YL, Hsu CN (2024) Melatonin Use during Pregnancy and Lactation Complicated by Oxidative Stress: Focus on Offspring’s Cardiovascular-Kidney-Metabolic Health in Animal Models. Antioxid (Basel) 13(2):226. 10.3390/antiox13020226 PMC10886428 38397824

[56] Alves MS, Damous LL, Ferreira IDS, Ferreira CDS, Sorpreso ICE, Cipolla-Neto J, Baracat EC, Soares JM Jr. (2026) Melatonin’s oncostatic effects in experimental breast cancer: A systematic review of dose-response and apoptotic mechanisms in animal models. Clin (Sao Paulo) 81:100888. 10.1016/j.clinsp.2026.100888 PMC12955072 41740526

[57] Gandolfi JV, Di Bernardo APA, Chanes DAV, Martin DF, Joles VB, Amendola CP, Sanches LC, Ciorlia GL, Lobo SM (2020) The Effects of Melatonin Supplementation on Sleep Quality and Assessment of the Serum Melatonin in ICU Patients: A Randomized Controlled Trial. Crit Care Med 48(12):e1286–e1293. 10.1097/CCM.0000000000004690 33048904

[58] Abdelrahman AMF, Omara AFAS, Elzohry AAM (2020) Safety and Efficacy of Oral Melatonin When Combined with Thoracic Epidural Analgesia in Patients with Bilateral Multiple Fracture Ribs. Local Reg Anesth 13:21–28. 10.2147/LRA.S244510 32341662 PMC7166071

[59] Tinto M, Sartori M, Pizzi I, Verga A, Longoni S (2020) Melatonin as host modulating agent supporting nonsurgical periodontal therapy in patients affected by untreated severe periodontitis: A preliminary randomized, triple-blind, placebo-controlled study. J Periodontal Res 55(1):61–67. 10.1111/jre.12686 31407353

[60] Garofoli F, Longo S, Pisoni C, Accorsi P, Angelini M, Aversa S, Caporali C, Cociglio S, De Silvestri A, Fazzi E, Rizzo V, Tzialla C, Zecca M, Orcesi S (2021) Oral melatonin as a new tool for neuroprotection in preterm newborns: study protocol for a randomized controlled trial. Trials 22(1):82. 10.1186/s13063-021-05034-w 33482894 PMC7820522

[61] Marseglia L, Gitto E, Laschi E, Giordano M, Romeo C, Cannavò L, Toni AL, Buonocore G, Perrone S (2021) Antioxidant Effect of Melatonin in Preterm Newborns. Oxid Med Cell Longev 2021:6308255. 10.1155/2021/6308255 34840669 PMC8626170

[62] Lozano A, Marruecos J, Rubió J, Farré N, Gómez-Millán J, Morera R, Planas I, Lanzuela M, Vázquez-Masedo MG, Cascallar L, Giralt J, Escames G, Valentí V, Grima P, Bosser R, Tarragó C, Mesía R (2021) Randomized placebo-controlled phase II trial of high-dose melatonin mucoadhesive oral gel for the prevention and treatment of oral mucositis in patients with head and neck cancer undergoing radiation therapy concurrent with systemic treatment. Clin Transl Oncol 23(9):1801–1810. 10.1007/s12094-021-02586-w 33738704

[63] Hoseini SG, Heshmat-Ghahdarijani K, Khosrawi S, Garakyaraghi M, Shafie D, Roohafza H, Mansourian M, Azizi E, Gheisari Y, Sadeghi M (2021) Effect of melatonin supplementation on endothelial function in heart failure with reduced ejection fraction: A randomized, double-blinded clinical trial. Clin Cardiol 44(9):1263–1271. 10.1002/clc.23682 34184295 PMC8427988

[64] Hoseini SG, Heshmat-Ghahdarijani K, Khosrawi S, Garakyaraghi M, Shafie D, Mansourian M, Roohafza H, Azizi E, Sadeghi M (2022) Melatonin supplementation improves N-terminal pro-B-type natriuretic peptide levels and quality of life in patients with heart failure with reduced ejection fraction: Results from MeHR trial, a randomized clinical trial. Clin Cardiol 45(4):417–426. 10.1002/clc.23796 35170783 PMC9019884

[65] Lan SH, Lee HZ, Chao CM, Chang SP, Lu LC, Lai CC (2022) Efficacy of melatonin in the treatment of patients with COVID-19: A systematic review and meta-analysis of randomized controlled trials. J Med Virol 94(5):2102–2107. 10.1002/jmv.27595 35032042 PMC9015545

[66] García-García I, Seco-Meseguer E, Ruiz-Seco P, Navarro-Jimenez G, Martínez-Porqueras R, Espinosa-Díaz M, Ortega-Albás JJ, Sagastagoitia I, García-Morales MT, Jiménez-González M, Martínez de Soto L, Bajo-Martínez AI, Del Palacio-Tamarit M, López-García R, Díaz-García L, Queiruga-Parada J, Giesen C, Pérez-Villena A, de Castro-Martínez M, González-García JJ, Rodriguez-Rubio M, de la Oliva P, Arribas JR, Carcas AJ, Borobia AM (2022) Melatonin in the Prophylaxis of SARS-CoV-2 Infection in Healthcare Workers (MeCOVID): A Randomised Clinical Trial. J Clin Med 11(4):1139. 10.3390/jcm11041139 35207411 PMC8876218

[67] Ameri A, Frouz Asadi M, Ziaei A, Vatankhah M, Safa O, Kamali M, Fathalipour M, Mahmoodi M, Hassanipour S (2023) Efficacy and safety of oral melatonin in patients with severe COVID-19: a randomized controlled trial. Inflammopharmacology 31(1):265–274. 10.1007/s10787-022-01096-7 36401728 PMC9676876

[68] Baradari AG, Habibi MR, Aarabi M, Sobhani S, Babaei A, Emami Zeydi A, Ghayoumi F (2022) The Effect of Preoperative Oral Melatonin on Postoperative Pain after Lumbar Disc Surgery: A Double-Blinded Randomized Clinical Trial. Ethiop J Health Sci 32(6):1193–1202. 10.4314/ejhs.v32i6.17 36475244 PMC9692143

[69] Holm MA, Gram-Hanssen A, Madsen BK, Zetner DB, Rosenberg J (2022) Oral melatonin did not reduce anxiety before elective hernia repair: A randomised, double-blinded, placebo-controlled trial. Acta Anaesthesiol Scand 66(9):1091–1098. 10.1111/aas.14128 36106858

[70] Rosas-Luna LE, Castelán-Martínez OD, Mora-Magaña I, Ángeles-Castellanos M, Ubaldo-Reyes LM (2022) Midazolam reduction with pre-operative melatonin in abdominal hysterectomy: double-blind randomized clinical trial. Cir Cir 90(3):353–358 English. 10.24875/CIRU.20001428 35636937

[71] Puliappadamb HM, Maiti R, Mishra A, Jena M, Mishra BR (2022 Summer) Efficacy and Safety of Melatonin as Prophylaxis for Migraine in Adults: A Meta-analysis. J Oral Facial Pain Headache 36(3–4):207–219. 10.11607/ofph.3211 PMC10586587 36445912

[72] Rigatto MH, Bergo P, Baldissera G, Beck E, David L, Santoro L, Barros A, Zanin R, Budelon Gonçalves JI, Falci D, Caumo W, Zavascki AP (2023) Melatonin for prevention of acute kidney injury in patients treated with intravenous polymyxin B: a double-blind, placebo-controlled randomized clinical trial. Clin Microbiol Infect 29(5):623–628. 10.1016/j.cmi.2022.12.017 36586514

[73] Rajan S, Abubaker R, Kala RA, Sasikumar NK, Kannan MV, Kumar L (2023 Oct-Dec) Effects of oral melatonin premedication on hemodynamic responses to intubation, anesthetic requirements and postoperative sedation: A randomized trial. J Anaesthesiol Clin Pharmacol 39(4):596–602. 10.4103/joacp.joacp_159_22 PMC10805198 38269180

[74] Quach D, Mol BW, Springer J, Tully E, Higgins C, Jones M, Hennes D, Pham Y, Swarnamani K, Palmer K, Davies-Tuck M (2024) A double-blind, randomized, placebo-controlled trial of melatonin as an adjuvant agent for induction of labor: The MILO trial. Acta Obstet Gynecol Scand 103(12):2442–2454. 10.1111/aogs.14951 39446608 PMC11609988

[75] Hannemann J, Laing A, Middleton B, Schwedhelm E, Marx N, Federici M, Kastner M, Skene DJ, Böger R (2024) Effect of oral melatonin treatment on insulin resistance and diurnal blood pressure variability in night shift workers. A double-blind, randomized, placebo-controlled study. Pharmacol Res 199:107011. 10.1016/j.phrs.2023.107011 38029806

[76] Menczel Schrire Z, Phillips CL, Duffy SL, Marshall NS, Mowszowski L, La Monica HM, Stranks L, Gordon CJ, Chapman JL, Saini B, Naismith SL, Grunstein RR, Hoyos CM (2024) 3-Month Melatonin Supplementation to Reduce Brain Oxidative Stress and Improve Sleep in Mild Cognitive Impairment: A Randomised Controlled Feasibility Trial. J Pineal Res 76(8):e70019 39702983 10.1111/jpi.70019

[77] Lange PW, Turbić A, Soh CH, Clayton-Chubb D, Lim WK, Conyers R, Watson R, Maier AB (2024) Melatonin does not reduce delirium severity in hospitalized older adults: Results of a randomized placebo-controlled trial. J Am Geriatr Soc 72(6):1802–1809. 10.1111/jgs.18825 38438279

[78] Ashokkumar P, Kuppusamy SK, Chinnasamy R (2024) Effects of melatonin on postoperative pain and sensory recovery following zygomaticomaxillary complex fractures - A randomized controlled trial. J Craniomaxillofac Surg 52(6):786–791. 10.1016/j.jcms.2024.03.037 38609755

[79] Holanda IRM, de Almeida Corrêa Alfredo M, Cassiano DP, Esposito ACC, Lima PB, Bagatin E, Miot HA (2024) Efficacy of oral 5 mg melatonin in the treatment of facial melasma in women: A double-blind, randomized, placebo-controlled clinical trial. J Eur Acad Dermatol Venereol 38(7):e607–e609. 10.1111/jdv.19784 38226560

[80] Rahafard S, Akbari Jokar Z, Hosseini SA, Alaee E (2024) The impact of oral melatonin on pain and anxiety reduction during venipuncture in pediatric patients: a double-blind randomized clinical trial. Ann Med Surg (Lond) 86(10):5811–5816. 10.1097/MS9.0000000000002163 39359816 PMC11444558

[81] Khaled M, Chui J, Ewusie J, Agzarian J, Bogach J, Gomez M, Shayegan B, Shargall Y, Alkhamesi N, Paul J, Thabane L, Shanthanna H (2025) Melatonin for preventing postoperative delirium in elderly patients: A multicenter randomized placebo-controlled pilot study. Med (Baltim) 104(8):e41615. 10.1097/MD.0000000000041615 PMC11856979 39998812

[82] Piriyakhuntorn P, Tantiworawit A, Phimphilai M, Kaewchur T, Niprapan P, Srivichit B, Apaijai N, Shinlapawittayatorn K, Chattipakorn N, Chattipakorn SC (2025) Melatonin Supplementation Alleviates Bone Mineral Density Decline and Circulating Oxidative Stress in Iron-Overloaded Thalassemia Patients. J Pineal Res 77(3):e70055. 10.1111/jpi.70055 40329508

[83] Farhadi K, Rahimi M, Varpaei H, Rajabi E, Tafreshi SMMT, Mohammadi P, Mohammadi M (2025) Ketamine and melatonin for the prevention of postoperative delirium in patients with colorectal cancer: a feasibility study. Perioper Med (Lond) 14(1):90. 10.1186/s13741-025-00571-3 40846975 PMC12372360

[84] Ruppel C, Rosa HH, Cardoso RB, Rigo NM, Soto VC, Bortoluzzi MC (2025) Preoperative 15 mg of melatonin for surgical discomfort, pain, edema and trismus in mandibular third molar surgery: a randomized double-blind placebo-controlled clinical trial. Clin Oral Investig 29(12):571. 10.1007/s00784-025-06649-y 41243001

[85] Behura SS, Panda SK, Tripathy S, Kujur I, Sahoo S, Pradhan DD (2025) Oral Melatonin for Pain Relief During Venepuncture in Neonates- a Pilot Randomized Control Trial. Eur J Clin Pharmacol 81(12):1787–1793. 10.1007/s00228-025-03914-7 40900317

[86] Larki RA, Iranmanesh A, Gholami D, Manzouri L (2025) The effect of oral melatonin on the quality of life, sleep and blood pressure of hemodialysis patients: a randomized clinical trial. BMC Nephrol 26(1):451. 10.1186/s12882-025-04387-7 40790573 PMC12341247

[87] Sánchez-García M, Tresguerres JAF, Álvarez-González M, Hera B, Puebla V, Ybañez L, Martínez-Sesmero JM, Blesa A, Martín-Benítez JC, Martínez-Sagasti F, González-Arenas P, Domingo S, Pardo C, Maichle S, Postigo C, De-Miguel S, Bringas M, González-Casanova R, Yordanov V, Delgado-Iribarren A, Culebras E, la-Hoz MAA (2026) Núñez-Reiz A. Oral Melatonin in Critically Ill Patients With COVID-19: A Quasi-Experimental Pragmatic Trial. J Med Virol 98(1):e70807. 10.1002/jmv.70807 41532840 PMC12802531

[88] Mundada KGP, Zambani S, Joshi J, Khaire T, Pawar P, Shelke S (2026) Oral Melatonin Supplementation for Sleep Disturbances in Children with Cerebral Palsy: A Randomized Double-Blind Controlled Trial. Indian J Pediatr 93(3):259–264. 10.1007/s12098-025-05873-4 41369870

[89] Nogueira PF, Vallim JRS, Barboza MNC, Andersen ML, Teixeira SH, Gracitelli CPB, Paranhos A Jr. Effects of Melatonin Supplementation on Sleep Quality in Patients with Advanced Glaucoma: A Randomized, Double-Masked, Placebo-Controlled Crossover Trial. Ophthalmol Glaucoma 2025 Dec 11:S2589-4196(25)00250-9. doi: 10.1016/j.ogla.2025.12.003 10.1016/j.ogla.2025.12.003 41386535

[90] Abdolrahim-Kashi E, Sadeghi H, Shafagh S, Biuki NM, Abed A, Moosavi GA (2026) Melatonin Supplementation Improves Pain, Sleep Quality, and Total Plasma Antioxidant Capacity in Women with Fibrocystic Breast Disease: A Randomized, Double-blind, Placebo-controlled Trial. J Res Pharm Pract 15(1):6. 10.4103/jrpp.jrpp_63_25 41969592 PMC13067964

[91] Al Alawi AM, Al Busaidi S, Al Rasbi SK, Al Farsi RS, Al Zeedy K, Al Huraizi AR, Al-Maqbali JS (2026) Effect of melatonin versus placebo for the prevention of delirium among medically hospitalised older patients: a double-blinded randomised controlled trial (project RESTORE). BMJ Open 16(1):e107775. 10.1136/bmjopen-2025-107775 41592826 PMC12853498

[92] Bejarano I, Jiménez-Jorge S, Lobo-Acosta MÁ, Álvarez-López AI, Rosso-Fernández CM, López-Ruiz R, Eichau S, Ruiz-Peña JL, Géniz MÁ, Ampuero J, Ponce-España E, Merino-Bohorquez V, Cameán M, García-Sánchez MI, Izquierdo G, Guerrero JM, Romero-Gómez M, Lardone PJ, Carrillo-Vico A (2026) Hepatic Safety of Adjunctive High-Dose Melatonin in Participants Receiving Ocrelizumab for Primary Progressive Multiple Sclerosis: Liver Toxicity Findings from a Phase I/II Randomised Clinical Trial (MELATOMS-1). CNS Drugs 40(4):579–596. 10.1007/s40263-025-01261-w 41706381 PMC12988967

[93] Abuhassan Q, Ghnim ZS, Mahdi MS, Kyada A, Abu Bakar H, Khare N, Singh D, Kubaev A, Taher WM, Alwan M, Jawad MJ, Hamad AK (2026) The effect of melatonin supplementation on lipid profile, oxidative stress, inflammatory marker, and sleep quality in patients with chronic kidney disease: a GRADE assessed meta-analysis. Front Nutr 13:1772877. 10.3389/fnut.2026.1772877 41727206 PMC12920205

[94] Andersen LP (2016) The analgesic effects of exogenous melatonin in humans. Dan Med J 63(10):B5289 27697139

[95] Rodríguez-Rubio M, Figueira JC, Acuña-Castroviejo D, Borobia AM, Escames G, de la Oliva P (2020) A phase II, single-center, double-blind, randomized placebo-controlled trial to explore the efficacy and safety of intravenous melatonin in patients with COVID-19 admitted to the intensive care unit (MelCOVID study): a structured summary of a study protocol for a randomized controlled trial. Trials 21(1):699. 10.1186/s13063-020-04632-4 32758298 PMC7403786

[96] Garioud ALB, Andersen LPK, Jensen AKG, Do HQ, Jakobsen JC, Holst LB, Rasmussen LS, Afshari A (2024) Intravenous MELAtonin for prevention of Postoperative Agitation and Emergence Delirium in children (MELA-PAED): A protocol and statistical analysis plan for a randomized clinical trial. Acta Anaesthesiol Scand 68(2):280–286. 10.1111/aas.14342 37904610

[97] Lalanne S, Fougerou-Leurent C, Anderson GM, Schroder CM, Nir T, Chokron S, Delorme R, Claustrat B, Bellissant E, Kermarrec S, Franco P, Denis L, Tordjman S (2021) Melatonin: From Pharmacokinetics to Clinical Use in Autism Spectrum Disorder. Int J Mol Sci 22(3):1490. 10.3390/ijms22031490 33540815 PMC7867370

[98] Reiter RJ, Mayo JC, Tan DX, Sainz RM, Alatorre-Jimenez M, Qin L (2016) Melatonin as an antioxidant: under promises but over delivers. J Pineal Res 61(3):253–278. 10.1111/jpi.12360 27500468

[99] Cruz-Sanabria F, Bruno S, Crippa A, Frumento P, Scarselli M, Skene DJ, Faraguna U (2024) Optimizing the Time and Dose of Melatonin as a Sleep-Promoting Drug: A Systematic Review of Randomized Controlled Trials and Dose-Response Meta-Analysis. J Pineal Res 76(5):e12985. 10.1111/jpi.12985 38888087

[100] Iyer S, Monk V, Slater R, Baxter L (2026) Exogenous Melatonin and Sleep Quality: A Scoping Review of Systematic Reviews. J Clin Pharmacol 66(2):e70115. 10.1002/jcph.70115 41014554 PMC12859178

[101] Salanitro M, Wrigley T, Ghabra H, de Haan E, Hill CM, Solmi M, Cortese S (2022) Efficacy on sleep parameters and tolerability of melatonin in individuals with sleep or mental disorders: A systematic review and meta-analysis. Neurosci Biobehav Rev 139:104723. 10.1016/j.neubiorev.2022.104723 35691474

[102] Liu R, Luo X, Li J, Lei Y, Zeng F, Huang X, Lan Y, Yang F (2022) Melatonin: A window into the organ-protective effects of sepsis. Biomed Pharmacother 154:113556. 10.1016/j.biopha.2022.113556 35994818

[103] Xu J, Liang C, Yao S, Wang F (2025) Melatonin Exerts Positive Effects on Sepsis Through Various Beneficial Mechanisms. Drug Des Devel Ther 19:1333–1345. 10.2147/DDDT.S509735 40026332 PMC11871935

[104] Zetner D, Andersen LP, Rosenberg J (2016) Pharmacokinetics of Alternative Administration Routes of Melatonin: A Systematic Review. Drug Res (Stuttg) 66(4):169–173. 10.1055/s-0035-1565083 26514093

